# Unlocking the potential of high-resolution multimodality neuromonitoring for traumatic brain injury management: lessons and insights from cases, events, and patterns

**DOI:** 10.1186/s13054-025-05360-4

**Published:** 2025-03-31

**Authors:** Stefan Yu Bögli, Erta Beqiri, Ihsane Olakorede, Marina Sandra Cherchi, Claudia Ann Smith, Xuhang Chen, Guido Di Tommaso, Tommaso Rochat, Masumi Tanaka Gutiez, Giada Cucciolini, Virginia Motroni, Adel Helmy, Peter Hutchinson, Andrea Lavinio, Virginia F. J. Newcombe, Peter Smielewski

**Affiliations:** 1 Brain Physics Laboratory, Division of Neurosurgery, Department of Clinical Neurosciences, Addenbrookes Hospital, University of Cambridge, Cambridge, UK; 2 Division of Neurosurgery, Department of Clinical Neurosciences, University of Cambridge, Cambridge, UK; 3 Division of Anaesthesia, Department of Medicine, University of Cambridge, Cambridge, UK; 4 Department of Medicine, University of Cambridge, Cambridge, UK; 5 Department of Neurology and Neurocritical Care Unit, Clinical Neuroscience Center, University Hospital Zurich, University of Zurich, Zurich, Switzerland; 6 Department of Critical Care, Marques de Valdecilla University Hospital, and Biomedical Research Institute (IDIVAL), Santander, Cantabria Spain; 7 Department of Surgical, Medical, Molecular Pathology and Critical Care Medicine, University of Pisa, Pisa, Italy; 8 Grande Ospedale Metropolitano Niguarda, Milan, Italy; 9 Intensive Care Unit, University Hospital of Geneva, Geneva, Switzerland; 10 Departmental Structure of Neuroanesthesia and Critical Care, Azienda Ospedaliero-Universitaria Pisana, Pisa, Italy

**Keywords:** Multimodality neuromonitoring, Traumatic brain injury, Education, Patient management

## Abstract

Multimodality neuromonitoring represents a crucial cornerstone for patient management after acute brain injury. Despite the potential of multimodality neuromonitoring (particularly high-resolution neuromonitoring data) to transform care, its full benefits are not yet universally realized. There remains a critical need to integrate the interpretation of complex patterns and indices into the real-time clinical decision-making processes. This requires a multidisciplinary approach, to evaluate and discuss the implications of observed patterns in a timely manner, ideally in close temporal proximity to their occurrence. Such a collaborative effort could enable clinicians to harness the full potential of multimodal data. In this educational case-based scoping review, we aim to provide clinicians, researchers, and healthcare professionals with detailed, compelling examples of potential applications of multimodality neuromonitoring, focused on high-resolution modalities within the field of traumatic brain injury. This case series showcases how neuromonitoring modalities such as intracranial pressure, brain tissue oxygenation, near-infrared spectroscopy, and transcranial Doppler can be integrated with cerebral microdialysis, neuroimaging and systemic physiology monitoring. The aim is to demonstrate the value of a multimodal approach based on high-resolution data and derived indices integrated in one monitoring tool, allowing for the improvement of diagnosis, monitoring, and treatment of patients with traumatic brain injury. For this purpose, key concepts are covered, and various cases have been described to illustrate how to make the most of this advanced monitoring technology.

**Supplementary Information:**

The online version contains supplementary material available at 10.1186/s13054-025-05360-4.

## Introduction

**Fig. 1:**
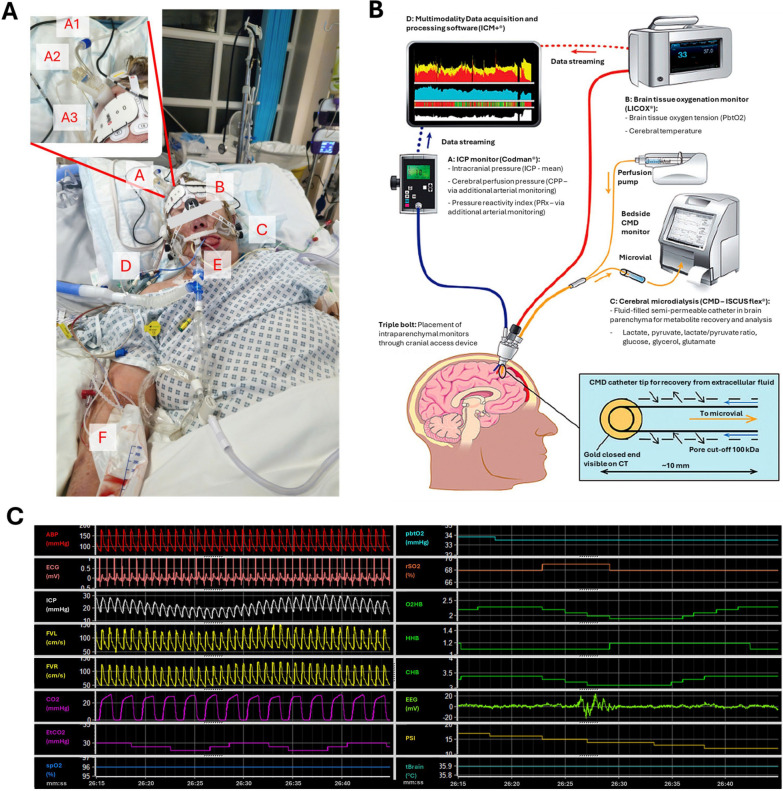
Multimodality monitoring setup**.** The full monitoring setup applied is displayed in panel **A**. The following devices (and derived parameters) are used for monitoring: A—invasive neuromonitoring modalities—A1 cerebral microdialysis, A2 ICP monitor, A3 PbtO2 and temperature monitor (the derived parameters are described in panel **B**); B—NIRS (rSO2, oxygenated and deoxygenated hemoglobin fractions) and electroencephalography (EEG, incl. raw traces, patient state index) patches; C—TCD (FV); D—central jugular catheter (central venous pressure); E—tracheal tube (capnography, EtCO2, respiratory rate), and esophageal temperature probe (core temperature); F—arterial line (ABP) and finger pulse oximeter (spO2). Panel **B** describes the methodology and devices used for invasive monitoring which entails the insertion of a multi-channel cranial access device (i.e., triple bolt—allows for the combined insertion and securing of the different invasive probes). The figure used in panel **B** was adapted from the original version published by Khellaf et al.[13] Panel **C** shows an excerpt of the various raw (i.e. unprocessed) high-resolution (often waveform level) data traces as collected within the ICM+ software

Multimodality neuromonitoring (MMM) represents a cornerstone of traumatic brain injury (TBI) intensive care management. [1] MMM refers to the integration of different modalities and metrics used for brain physiology monitoring [2]. This educational case-based review focuses on the utility and importance of high-resolution—i.e. waveform level—data [3] which can be acquired as part of MMM to allow for a deeper understanding of the dynamic processes that follow TBI. MMM allows for a real-time bedside depiction of these changes and enables the calculation of various indices. MMM combines routine intensive care unit (ICU) vital-sign monitoring metrics such as arterial blood pressure (ABP), heart rate (HR), core temperature, arterial blood oxygen saturation (spO2) and mechanical ventilation derived metrics including end-tidal CO2 (EtCO2), with different neuromonitoring specific metrics. These include intracranial pressure (ICP), brain tissue oxygenation (PbtO2), regional oxygen saturation (rSO2; derived from near infrared spectroscopy—NIRS), and at times even cerebral blood flow velocity (FV; from transcranial Doppler (TCD) monitoring). The setup used in our ICU that allows for the collection and integration of these various modalities is described in Fig. 1. ICP and CPP (cerebral perfusion pressure; mathematical difference between mean ABP and ICP) help manage intracranial hypertension and ensure adequate cerebral perfusion. PbtO2 and rSO2 monitoring provide important (albeit focal) information on brain and blood oxygen levels, which are critical for preventing hypoxic injury. TCD can be used to add information on FV, cerebrovascular autoregulation and other cerebrovascular properties [4]. When combined, these modalities provide the user with rich, additional information (e.g. the pressure reactivity index—PRx[5] or the compensatory reserve index—RAP[6]). However, some of these derived metrics require full waveform-level resolution, which at present requires additional 3rd party software [2, 7]. Continuous MMM should be supplemented by intermittent approaches like microdialysis [8] and cerebral imaging (e.g. CT, MRI), to allow for a more comprehensive understanding of the physiological state.

Despite the vast information that could be inferred from MMM, there is a large center-to-center variability in MMM use, due to insufficient consensus, lack of evidence, as well as high costs and time associated with its use [9]. The integration of these various modalities increases complexity and often requires greater expertise for interpretation. At times this might lead to increased uncertainty brought by the growing number of parameters to follow. Uncertainty itself is a growing issue within health care [10–12], and rises particularly if the measures display contradictory results (either due to device related limitations or the involvement of different mechanisms). This might not only hinder clinical interpretation but also draw attention away from the relevant key measures such as ICP or CPP.

In this educational case-based review of MMM modalities, we aim to provide clinicians, researchers, and healthcare professionals with detailed examples of how MMM helps improve the understanding of physiology after TBI with a particular focus on indices derived from high resolution physiological monitoring. The review consists of two distinct sections: In the first section, we describe the various modalities explaining the key concepts as well as the potential information that can be derived. In the second section, by integrating clinical cases, we aim to bring theoretical concepts closer to the reader, facilitating visualization and practical interpretation of information that can be derived from these monitoring modalities.

## Materials and methods

For this study, deidentified physiological monitoring data and clinical descriptors were accessed from the Brain Physics database (REC 23/YH/0085). Informed consent was waived by the local ethics committee. MMM is standard of care for TBI patients admitted to our Neurocritical Care Unit which lies within a Major Trauma Center (Addenbrooke’s Hospital, Cambridge, UK). Data was acquired from the various monitoring devices using the ICM + ® software (Cambridge Enterprise Ltd, Cambridge, UK). TCD was performed as part of a clinical audit (Clinical Project ID4201) [14]. Microdialysis and imaging data was collected as part of a separate research protocol (LREC 97/290). Clinical descriptors were extracted from the database or the electronic patient files as necessary. The outcome is described using the Glasgow Outcome Scale Extended [15]—GOSE—assessed at 6 months after ictus during outpatient consultations or via telephone interviews by trained staff.

The TBI management protocol, which largely follows the Brain Trauma Foundation guidelines has previously been described [16]. The ICP/CPP treatment protocol is shown in Supplement A. While the decision to initiate invasive MMM largely reflects the recommendations of the Brain Trauma Foundation [17] and the Milan consensus conference [18], the final decision remains responsibility of the treating team of physicians who consider each patient’s unique circumstances. Certainly, ICP monitoring is indicated for comatose TBI patients when sedation interruption is unsafe or the clinical examination is unreliable, after secondary decompressive craniectomy (DC), and following evacuation of an acute hematoma at elevated risk of intracranial hypertension. For invasive monitoring, a triple-lumen bolt (Fig. 1) is inserted to facilitate insertion of the intraparenchymal cerebral pressure monitor, and the cerebral microdialysis and PbtO2 probes.

## Section 1: Key concepts of multimodality neuromonitoring

In this section, we introduce the important measures that can be derived from MMM. We abstained from a separate introduction of ICP and CPP, as these have been extensively described [19]. The specific aspects covered within this section are: 1. Cerebrovascular reactivity—PRx; 2. The relationship between CPP and PRx—CPPopt (optimal CPP) and the lower limit of reactivity (LLR); 3. The autoregulation curve; 4. Cerebral compensatory reserve and brain compliance; 5. NIRS and rSO2; 6. PbtO2; 7. CO2 reactivity; 8. Cerebral microdialysis.

### Key concept 1: Cerebrovascular reactivity—PRx

Cerebral autoregulation describes a physiological mechanism by which the brain maintains constant CBF (cerebral blood flow) despite changes in CPP [20, 21]. PRx describes cerebrovascular reactivity and can be used as an proxy measure of cerebrovascular autoregulation [5]. PRx reflects the extent of arteriolar constriction or dilation in response to changes in ABP. It is calculated as a moving correlation coefficient between 10 s mean ABP and mean ICP values over consecutive 5-min time windows. Negative PRx values suggest intact cerebrovascular reactivity (i.e. ABP slow waves are adequately counteracted), while positive PRx values indicate impaired cerebrovascular reactivity, where an increase in ABP results in an increase in ICP due to the vessels failing to sufficiently counteract the change in ABP. Positive PRx values are accompanied by a higher risk of hyper- or hypoperfusion and an increased risk of intracranial hypertension. Similarly, impaired vascular reactivity is associated with worse outcome after TBI [22]. In addition, by exploring the relationship between PRx and CPP, CPP targets such as CPPopt or LLR (see Key Concept 2), that can be used to individualize CPP management can be identified [23, 24]. From a clinical standpoint, it is crucial to recognize that impaired cerebrovascular reactivity does not inherently result in compromised brain perfusion. Cerebrovascular reactivity reflects the brain’s ability to counteract dynamic changes in CBF, rather than directly indicating insufficient perfusion. If CPP is maintained within the range necessary to sustain cerebral metabolism and remains outside the thresholds associated with hypo- or hyperperfusion, cerebrovascular autoregulation and reactivity are not strictly required to prevent secondary brain injury. This does not void the importance of vigilant clinical monitoring and management in absence of functioning reactivity. Along the same lines, working autoregulation also does not preclude the presence or development of secondary brain injury.

### Key concept 2: the relationship between CPP and PRx—CPPopt and LLR

**Fig. 2:**
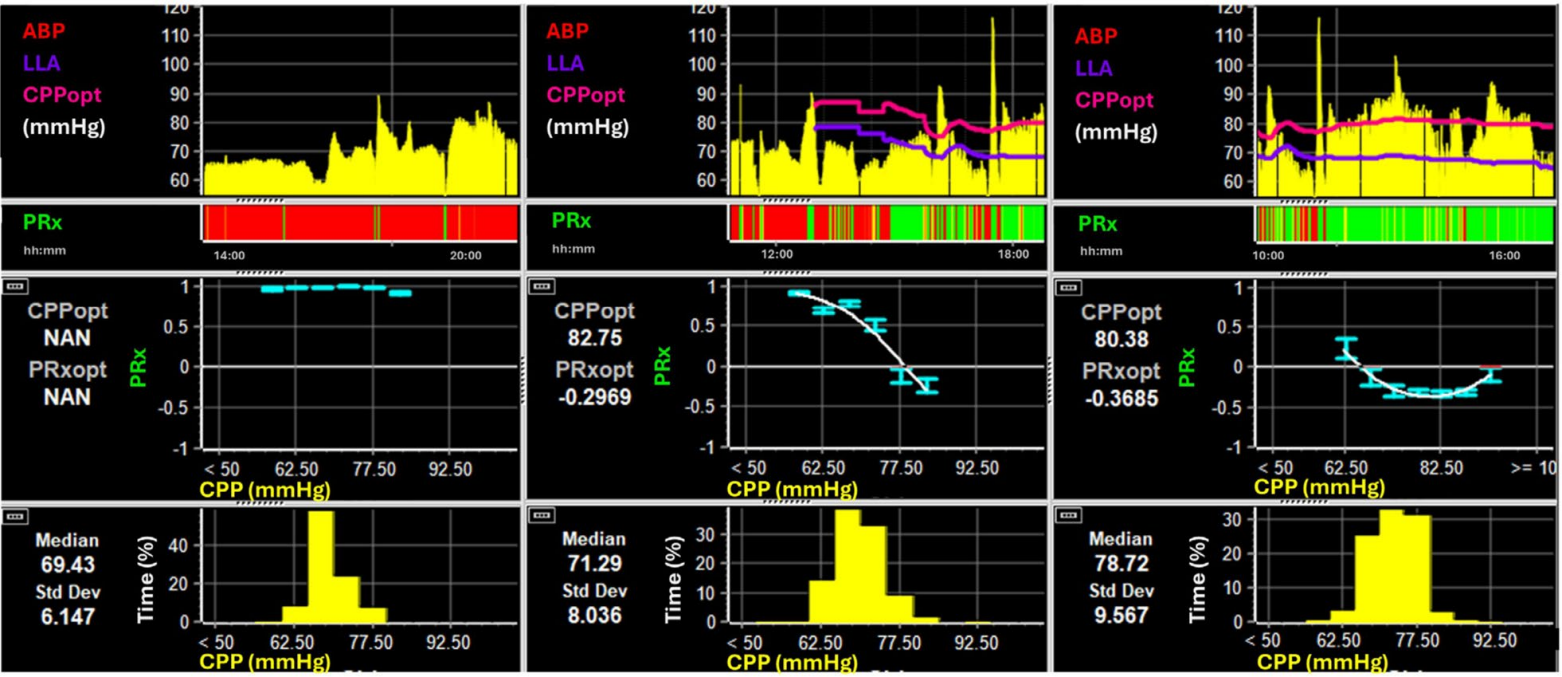
The relationship between CPP and PRx. Three 8-h sections with their respective CPP/PRx relationship plots are displayed. If available, the automated CPPopt (pink) and LLR (purple) time trends are superimposed on the raw CPP time trends. Of note, while the CPP/PRx plots describe the relationship over the whole data buffer considered, the time trends are computed as minute-by-minute calculations based on the previous data buffer, as explained in the text. When comparing the three sections, we can appreciate the importance of a sufficient spread of both PRx and CPP for the estimation of the CPP/PRx relationship. In the first section irrespective of CPP, PRx is outside of the range with intact reactivity. In the middle panel, we can appreciate the lower bounds of the relationship including the LLR (at around 70 mmHg). The full U-shape curve can only be appreciated in the panel on the right. Within this curve, CPPopt denotes the “optimal” CPP level (i.e. associated with the lowest PRx value) while PRxopt is defined as the PRx value that is associated with the optimal level of CPP (i.e. CPPopt). Of note, in the second panel, while the CPP/PRx plot did not show the full U-shape curve in the time period considered, the automated trends of both CPPopt and LLR could be assessed for some of the time. Details of the automated methods for assessment of CPPopt and LLR are out of scope in this manuscript and can be found elsewhere [27]. Lastly, while the time trends of CPPopt and LLR are often very similar, they can also differ widely, denoting the dynamic aspect of the range of vascular reactivity

In 2002 the concept of individualizing CPP based on PRx was introduced with the description of CPPopt (i.e. optimal CPP) [23]. It was established upon the observation of an often U-shaped relationship between corresponding values of CPP and PRx in population aggregated data. CPPopt is defined as the CPP at the lowest point of this curve. Time trends of CPPopt can be computed semi-continuously [25–27], by assessing the CPP/PRx relationship for individual patients using shorter sections of data (e.g. 2–8 h of data). While there are no prospective studies evaluating the utility of targeting CPP at CPPopt, such approach has been deemed safe and feasible[24] and CPPopt has been introduced to some medical devices (i.e. Moberg CNS Monitor, Raumedic NeuroSmart [28, 29]) in addition to ICM+ . The availability of CPPopt relies on the naturally occurring variations in CPP. For its valid estimation a sufficient spread of CPP and PRx is necessary, which is not always possible (e.g. in cases without changes in the state of reactivity—Fig. 2). A second index that can be derived from this relationship is the lower limit of reactivity—i.e. LLR. While CPPopt denotes the CPP value with optimal reactivity, the LLR represents the lower breakpoint below which reactivity worsens with further decreases in CPP. The concept is based on clinical findings describing an association between time spent below the LLR and worse outcome [30, 31] and clinical reasoning which aligns with physiological findings [32] (see key concept 3). Similarly, the upper limit of reactivity can be computed, but this limit has not been adequately investigated yet [33].

### Key concept 3: the autoregulation curve

**Fig. 3:**
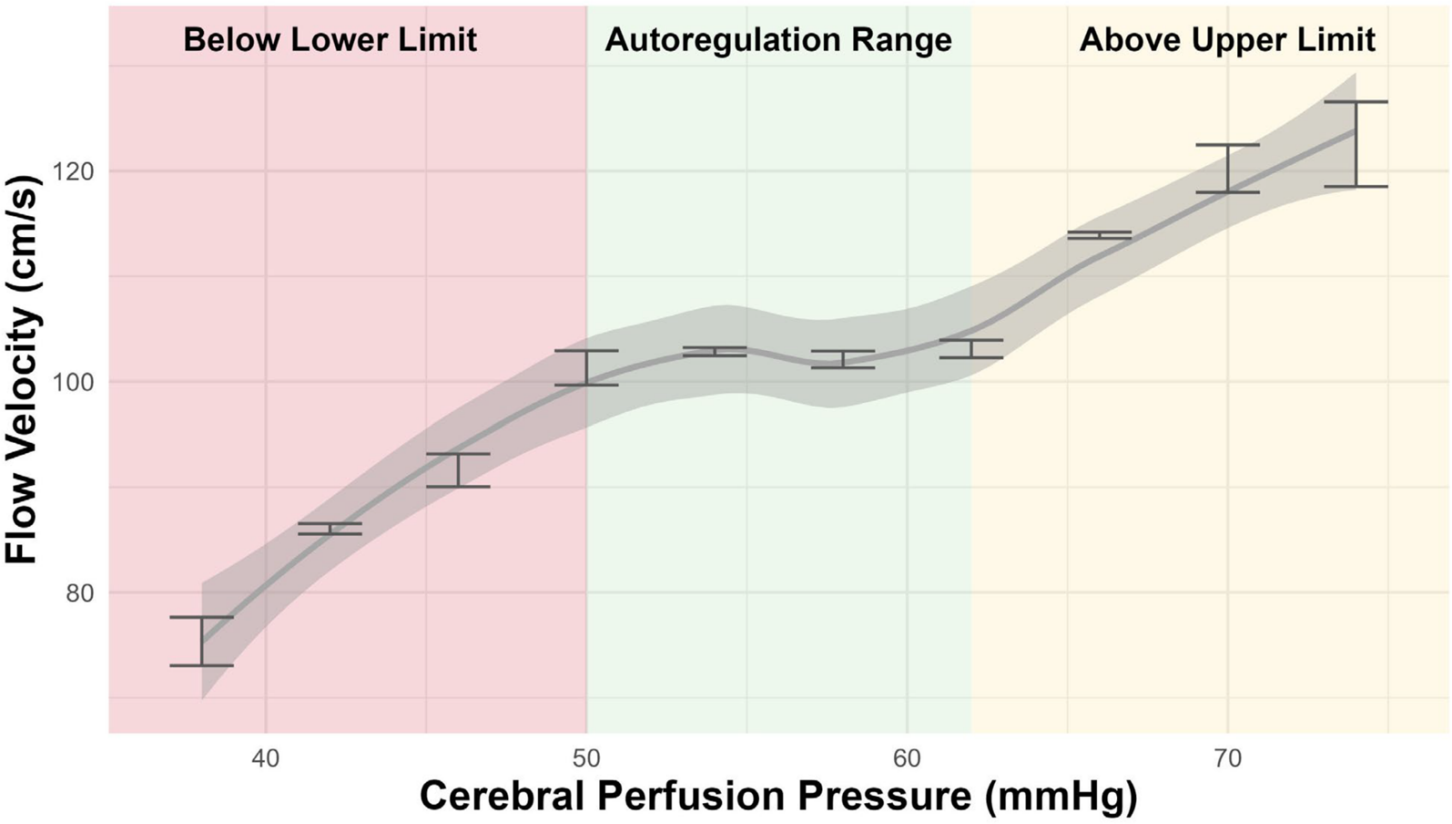
The Lassen autoregulation curve. This figure illustrates an example Lassen autoregulation curve from a single patient. It depicts the relationship between various levels of CPP and FV. For each CPP level, we plotted the standard errors as bars. The curve is estimated using a locally weighted scatterplot smoothing method along with the corresponding confidence interval (shaded region). The resulting autoregulation curve is divided into three distinct regions: (1) the autoregulation range (middle region), where cerebral perfusion remains stable despite fluctuations in CPP; (2) the upper limit of autoregulation (right segment), where increases in CPP result in corresponding increases in FV; and (3) the lower limit of autoregulation (left segment), where decreases in CPP lead to reductions in FV. The shape, steepness, and width are to an extent patient and disease specific

The autoregulation curve (Fig. 3), formally known as the “Lassen Curve”, describes the relationship between changes in CPP and CBF [20, 21]. It was introduced by N.A. Lassen in 1959 and describes how the brain maintains a stable blood flow despite changes in CPP [34]. The autoregulation curve is classically separated into three parts, namely the autoregulation range and the regions above the upper and below the lower limit of the autoregulation range. The autoregulation range corresponds to the mid-region of the curve, classically defined as the plateau, within which changes in CPP are optimally counteracted allowing for stable perfusion. At either end of this region there are the upper and lower break points, outside of which CBF cannot be stabilized optimally. Below the lower limit, CBF decreases linearly with decreasing CPP, leading to an increased risk of hypoperfusion. Above the upper limit, CBF increases linearly with increasing CPP which effectively leads to hyperperfusion. Both are associated with a higher risk for secondary brain injury [30, 31, 33]. It is important to note that during daily clinical practice, the lower break point is rarely visible since pressures are kept higher to omit the detrimental effects of hypoperfusion. Nevertheless, the lower limit has been shown to vary across patients and is dynamic in nature, and episodes with CPP below the lower limit can be detrimental [30]. While pushing CPP above the upper breakpoint is certainly also associated with negative effects such as increasing the chance for cerebral oedema, it seems to be less detrimental than the consequences of hypoperfusion [31].

**Fig. 4:**
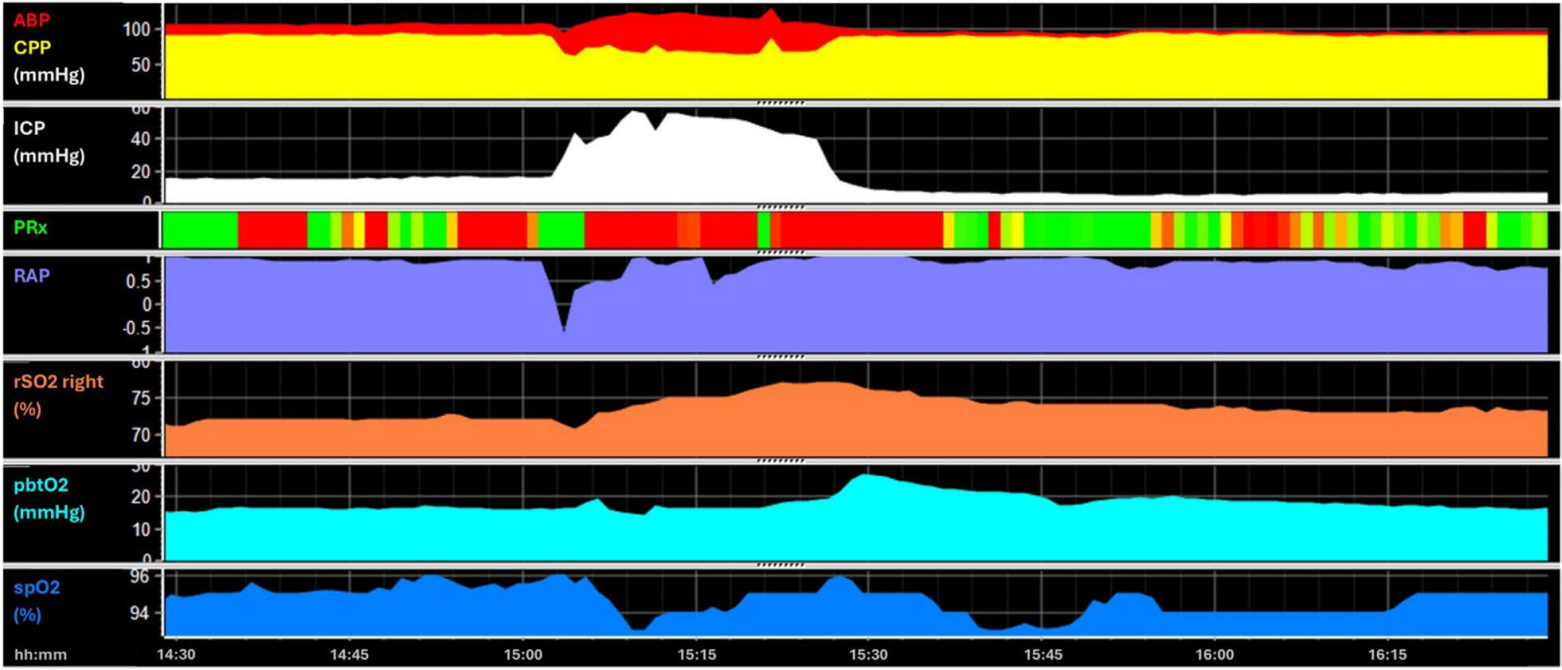
Example—plateau wave. A brief drop in CPP and ABP is followed by a distinct increase in ICP from around 18 mmHg to above 40 mmHg for 20 min. At the timepoint of the drop the patient displayed preserved cerebrovascular reactivity (PRx, represented with a color-coded bar, where red indicates impaired and green indicates preserved vascular reactivity) causing a rise in ICP which then initiates a vicious cycle of increasing cerebral blood volume (which can be appreciated via the proxy measure rSO2) and ICP which lead to a sustained decrease in CPP

To make matters more complex, fully functioning autoregulation itself may, paradoxically, play a role in detrimental sequelae such as plateau waves (Fig. 4). Plateau waves are sudden, sustained elevations in ICP that can occur in patients with acute brain injury including TBI [35]. They result from complex interactions between CBF, autoregulation, and intracranial compliance [36]. They are preceded by a vasodilatory trigger, most commonly a brief reduction of ABP. When cerebral autoregulation is functioning, the reduced ABP or CPP is followed by cerebral vasodilation causing a large increase in cerebral blood volume. In patients with reduced compliance this increase in volume translates to an increase in ICP which further decreases CPP consequently initiating a positive feedback loop increasing cerebral blood volume and ICP. Plateau waves are often sustained for 5–10 min before terminating abruptly either because of compensatory mechanisms (i.e. cerebrospinal fluid redistribution) or interventions (hyperventilation, cerebrospinal fluid drainage, osmotherapy etc.).

### Key concept 4: cerebral compensatory reserve and brain compliance

**Fig. 5:**
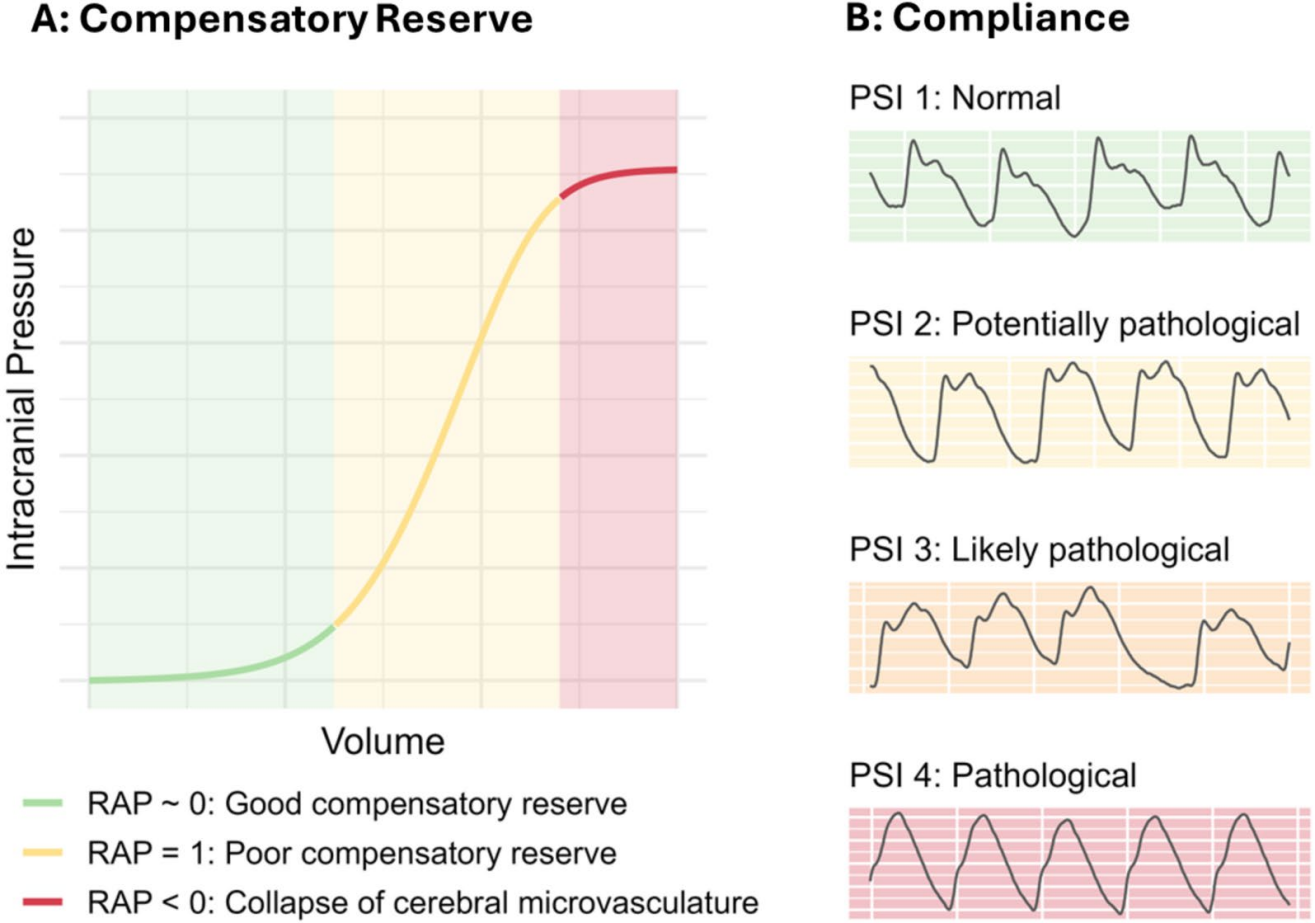
Cerebrospinal pressure volume curve and pulse shape index. Panel **A** displays the relationship between changes in volume and ICP. Depending on the state of the compensatory reserve (good vs. poor vs. collapse of cerebral microvasculature), the relationship between volume increases and ICP amplitude changes. When the compensatory reserve is high, the ICP amplitude does not alter vastly with changes in volume. With decreasing compensatory reserve, there appears a positive relationship between large increases in ICP amplitude with increases in volume (poor compensatory reserve). Lastly, an inverse relationship occurs, with decreases in ICP amplitude during increases in volume due to the collapse of the cerebral microvasculature. Panel **B** displays example sections depicting the different waveform shapes which PSI attempts to estimate. PSI 1 represents normal waveforms associated with good compliance, PSI 2 and 3 represent decreases in compliance, while PSI 4 represents critically decreased compliance. The colors are used to indicate the change between physiological and pathological. Even though these parameters are related, the state described by one does not directly suggest the state of the other

Cerebral compliance quantifies the brain's ability to accommodate increases in intracranial volume without relevant increases in ICP [37]. When compliance is high, ICP is minimally affected by changes in intracranial volume (e.g. due to bleeding, occlusive hydrocephalus, or neurovascular coupling related transient cerebrovascular bed engorgements). When compliance is low, even small changes in volume can lead to large changes in ICP. This concept can often be appreciated during late tier ICP therapy when removing single drops of cerebrospinal fluid (using external ventricular drains) leads to large decreases in ICP. While the presented measures necessitate invasive monitoring of ICP, novel non-invasive methods (e.g. the Brain4Care device) are being explored, potentially allowing for their use in a wider patient population [38, 39]. The RAP index (i.e. the correlation coefficient between mean ICP and ICP amplitude (assessed as the fundamental harmonic of the ICP pulse waveform)) quantifies the brains’ compensatory reserve [6] as a surrogate measure of brain compliance (Fig. 5A). A low RAP (close to 0) suggests good compensatory reserve while a high RAP (close to 1) indicates poor compensatory reserve. At high values of ICP, above the upper break point of the pressure volume curve, RAP can be negative, denoting a collapse of the cerebral vasculature. The pulse shape index (PSI) continuously quantifies ICP pulse waveforms, which are related to different cerebral compliance states (Fig. 5B) [40, 41]. The pulse shape is typically described with three peaks, P1 P2 and P3. The percussion wave P1 is generated by the incoming arterial pressure and is under physiological conditions the highest peak, followed by P2 (the tidal wave) which directly reflects the elastic properties of the brain tissue and consequently compliance, and lastly P3 (the dicrotic wave) caused by the closure of the aortic valve. When compliance decreases, P2 slowly increases in amplitude until ultimately surpassing P1 or even becoming the only visible peak [41]. The PSI index [40, 42] is calculated by classifying each pulse of the ICP waveform into five categories (from normal to pathological or artefactual), using a trained convolutional neuronal network and ResNet based deep learning model, and by providing the average category over 5 min of time, with calculations usually updated every minute.

### Key concept 5: near-infrared spectroscopy and regional oxygen saturation

**Fig. 6:**
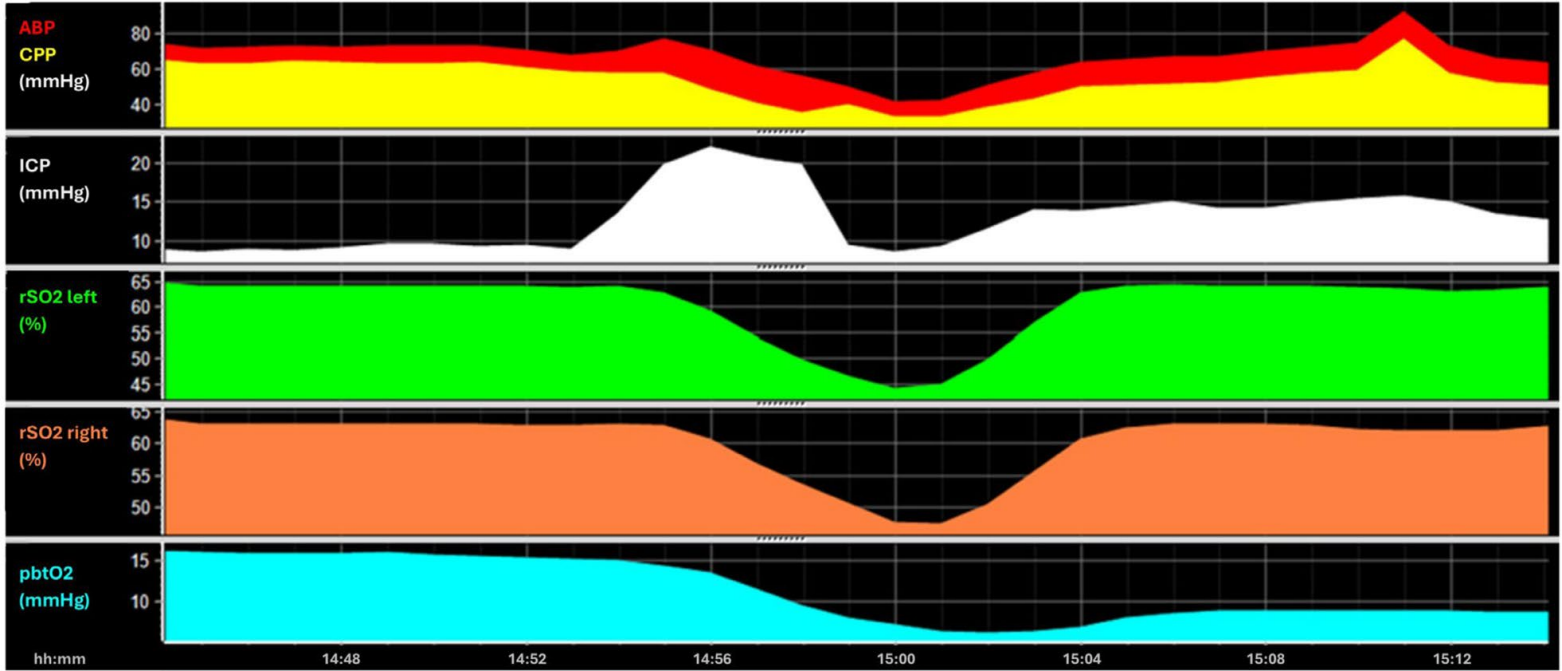
Example—effect of ICP on rSO2. ABP and CPP are displayed on the top above the ICP time trends. A distinct increase in ICP from below 10 mmHg to above 20 mmHg for 5 min representing a plateau wave can be appreciated. rSO2 left and right are shown in green and orange with PbtO2 displayed at the bottom. The increase in ICP was associated with a temporary decrease in CPP and consequently rSO2 and PbtO2. There is an earlier normalization of rSO2 after CPP increases to around 60 mmHg, while PbtO2 remains low

NIRS is a non-invasive, easily applied monitoring technique that assesses rSO2 usually within the frontal cortical region. rSO2 is provided as a percentage and is derived from changes in light-absorption by oxygenated and deoxygenated hemoglobin. The level of rSO2 depends on oxygen supply (blood flow) and demand (cellular oxygen consumption). Its accuracy and validity for the determination of intracerebral oxygenation remain to be determined [43]. Various factors affecting light transmission such as the extracranial soft tissue, the thickness of the skull, and external light sources hinder such validation [44–46]. Additionally, depending on the location and patient characteristics, different proportions in venous vs. arterial blood are covered. Yet, manufacturers use prespecified “expected” distributions of venous vs. arterial blood for the calculations [47]. In clinical practice, rSO2 is often seen as an easy to apply surrogate of TCD based flow measurements [48]. An example of the value of rSO2 is shown in Fig. 6, which depicts the change in rSO2 caused by an increase in ICP. Of note, the difference in impact of ICP on rSO2 compared to PbtO2 is clearly visible. rSO2 values return to baseline with normalization of CPP, while the PbtO2 remains impaired for an extended duration. Further differences between rSO2 and PbtO2 are discussed in Key Concept 6.

### Key concept 6: partial pressure of brain tissue oxygen—PbtO2

PbtO2 measures the partial pressure of oxygen within a small section of (usually frontal) white matter [49]. It requires the insertion of a probe into the brain’s parenchyma, usually placed in the vicinity of injured regions but within healthy (i.e. salvageable) tissue. Like rSO2, PbtO2 monitoring is particularly useful in TBI or other conditions wherein direct assessment of oxygen supply to specific brain regions (i.e. penumbral tissue) is critical. A decrease in PbtO2 has various potential causes such as reduced perfusion or oxygen supply, but also cerebral oedema or increases in ICP both of which lead to reduced CPP [50, 51]. There are distinct differences between PbtO2 and rSO2 [52]: 1. rSO2 provides an estimation of oxygenation in the superficial cortical regions, while PbtO2 measures the oxygenation within a certain depth within the white matter; 2. PbtO2 measures the partial pressure of all dissolved oxygen within the region, while rSO2 is directly dependent on the volume and relative saturations of hemoglobin. Consequently, increasing the fraction of inspired oxygen (e.g. when testing the PbtO2 probe) leads to a distinct increase in the measured PbtO2 while rSO2 increases minimally, since hemoglobin is often already saturated, but additional oxygen is dissolved within the blood.

### Key concept 7: CO2 reactivity

**Fig. 7:**
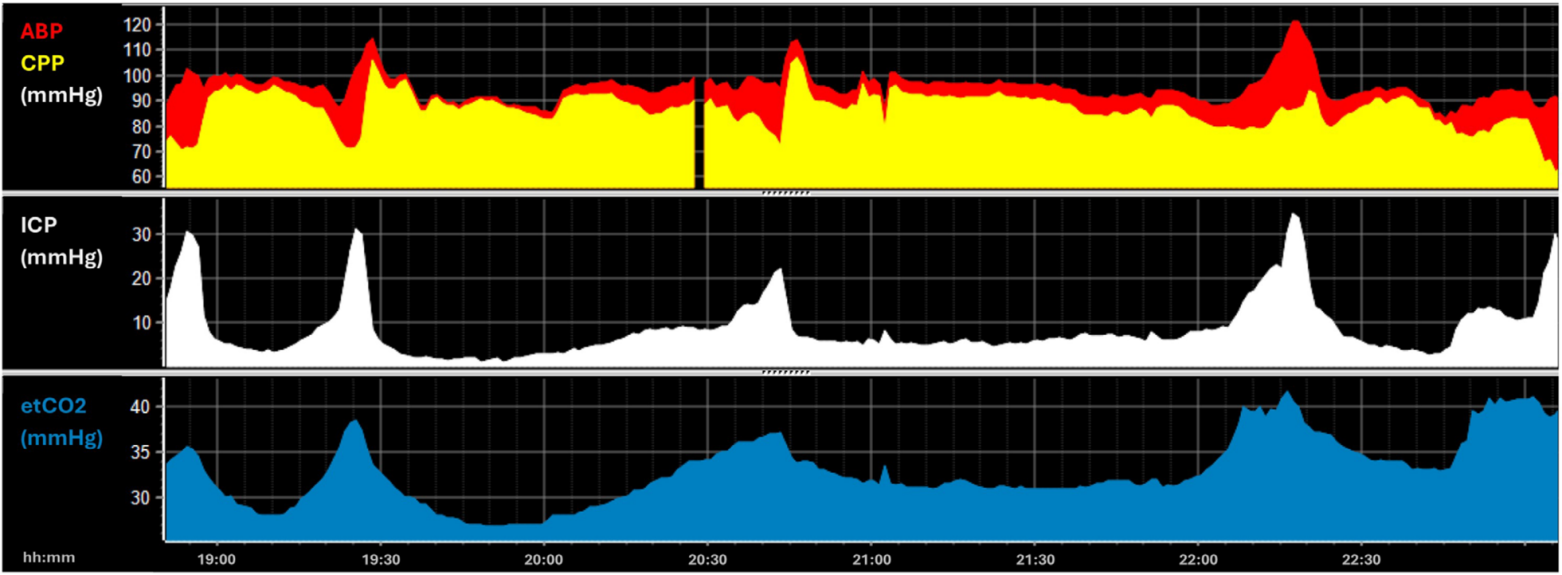
Example—CO2 reactivity. Distinct and concurrent decreases in CPP and increases in ICP are shown. When assessed in combination with the EtCO2 traces over an extended amount of time, it is evident that these resulted from CO2 reactivity with vasodilation in the cerebral vessels (and consequently cerebral blood volume and flow) with increasing EtCO2 levels

**Fig. 8:**
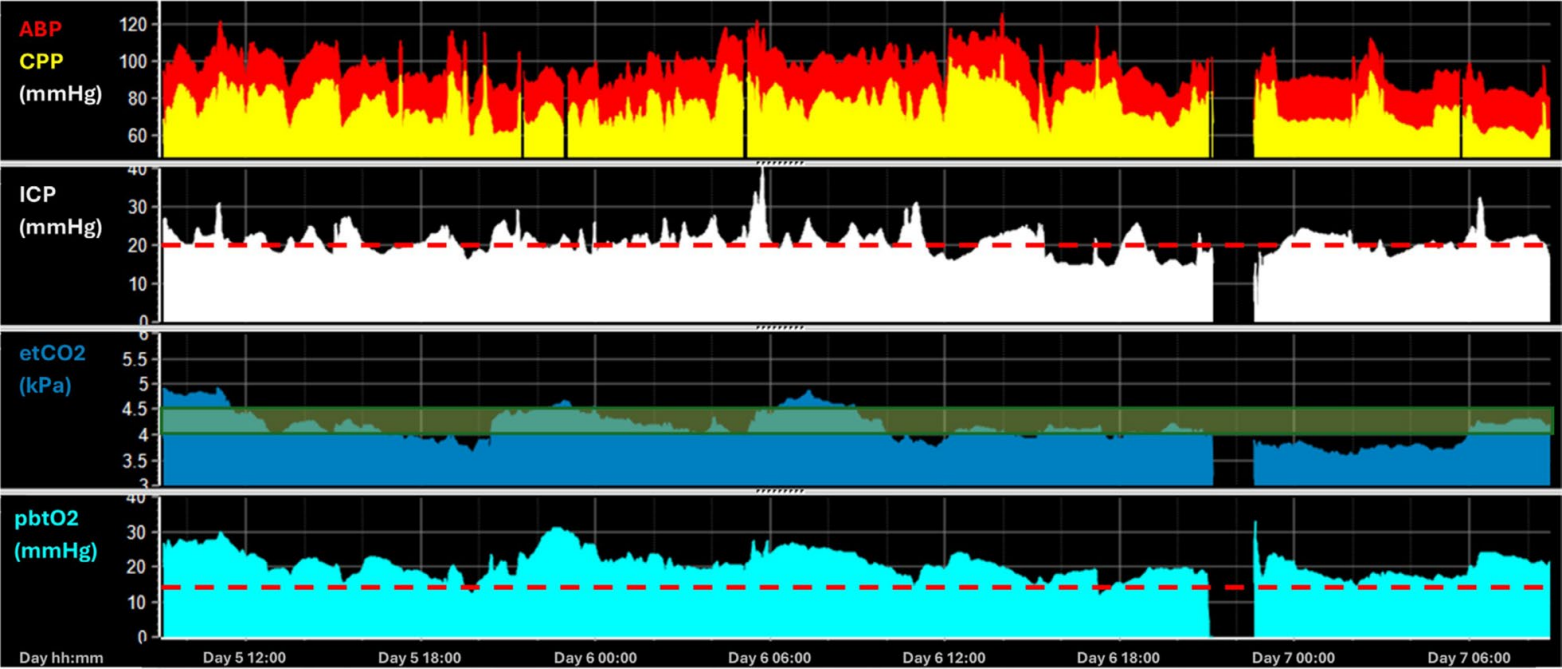
Example—PbtO2 monitoring for CO2 Management. In this patient, ICP was consistently close or above the treatment target of 20 mmHg (red dotted line superimposed on the ICP time trend). As part of the ICP-targeted management the patient received hyperventilation. The induced changes in EtCO2 (target range marked using a green box) caused a relatively concurrent change in PbtO2. In this case, even when EtCO2 was reduced below 4.0 kPa there was no period where PbtO2 dropped below 15 mmHg due to manipulation of other physiological parameters. This example illustrates how monitoring PbtO2 might ensure safety of moderate hyperventilation if required for emergent ICP control

CO2 reactivity refers to the brain's ability to regulate blood vessel diameters in response to changes in blood CO2 levels. In general, hypercapnia causes cerebral vasodilation (i.e. increase in CBF) while hypocapnia causes cerebral vasoconstriction [53]. This mechanism can be used to lower ICP, however after TBI, an alteration in CO2 reactivity (due to inflammation and altered neurovascular coupling) can occur [54]. In addition, the interplay between CO2 reactivity and vascular reactivity (see key concept 1) must be taken into consideration. The excerpt shown in Fig. 7 stems from a patient with recurrent episodes with intracranial hypertension which were counteracted with sedation and the administration of hypertonic saline without a sustained effect. Upon closer inspection of the multiple events, it is evident that each ICP-increase was preceded by an increase in EtCO2 which caused vasodilation. The opposing effect (vasoconstriction) can be appreciated in Fig. 8 which stems from a patient receiving moderate hyperventilation as part of the ICP-targeted therapy. When used for extended periods, hyperventilation carries the risk of ischemia, as well as the well-known rebound increase in ICP once hyperventilation is stopped. Reducing pCO₂ via hyperventilation lowers ICP by inducing vasoconstriction, which can have secondary impacts on cerebral oxygenation and metabolism [55], consequently PbtO2 might be promising for increasing the safety of CO2 management.

### Key concept 8: cerebral microdialysis—a semicontinuous monitoring method

Cerebral microdialysis is an invasive monitoring technique that provides semi-continuous insights into the brains metabolism [8]. Cerebral microdialysis allows for the measurement of key biochemical markers by sampling extracellular fluid of the brain parenchyma. For this purpose, a catheter with a semi-permeable membrane at its tip is inserted into brain tissue. A perfusion fluid is slowly pumped through the catheter. Small molecules such as glucose, lactate, pyruvate, glutamate, and glycerol diffuse across the membrane from the brain’s parenchyma into the perfusion fluid based on their concentration gradients. While the automated, continuous sampling solutions are being developed, the perfusates, now termed microdialysate, are collected and assessed on an hourly basis. These markers reflect various processes, including energy metabolism, ischemia, excitotoxicity, and cellular membrane integrity, and are, to some extent, representative of the brain’s metabolic state and of prognostic importance [56–58]. The most prominent derived marker is the lactate-to-pyruvate ratio, which quantifies the balance between aerobic and anaerobic metabolism. A high lactate-to-pyruvate ratio, often associated with low glucose levels, may indicate deranged cerebral physiology [59] or mitochondrial dysfunction [60]. Glucose on the other hand represents the primary energy substrate for the brain, while glutamate (a marker of excitotoxicity) and glycerol (a marker of cell membrane breakdown) represent markers of cellular injury. The hourly sampling interval means that each measurement reflects the metabolic activity over the preceding hour, rather than providing “real-time” information. Regardless, this advanced monitoring tool provides relatively high frequency neurochemistry data at the bedside compared to traditional analytical quantification methods.

## Section 2: case-based exploration

In this section, we transition from the theoretical framework of MMM described in section one to its application in real-world clinical scenarios. Through a series of cases, we aim to bridge the gap between textbook concepts and bedside practice, providing readers with a practical understanding of MMM for TBI management. The cases presented were developed retrospectively, and while some of the authors were part of the treating team, this inherently limits the ability to draw definitive conclusions about the impact of MMM on patient outcomes. Considering the often inconclusive or negative findings of large-scale studies evaluating MMM’s influence on clinical outcomes, this series is not intended to advocate for its routine use for improving patient’s outcome in any specific way. Instead, our primary goal is to highlight the potential of MMM as a tool for clarifying, understanding, or even deciphering the causes and consequences of various phenomena on the brain’s physiology. The main manuscript contains those cases where MMM directly influenced patient management and provided insights that may otherwise not have been evident through routine care alone. Additional interesting cases are included in the supplementary material, with only a brief summary provided here for reference.

### Case 1: Cerebrovascular reactivity-based management

**Fig. 9:**
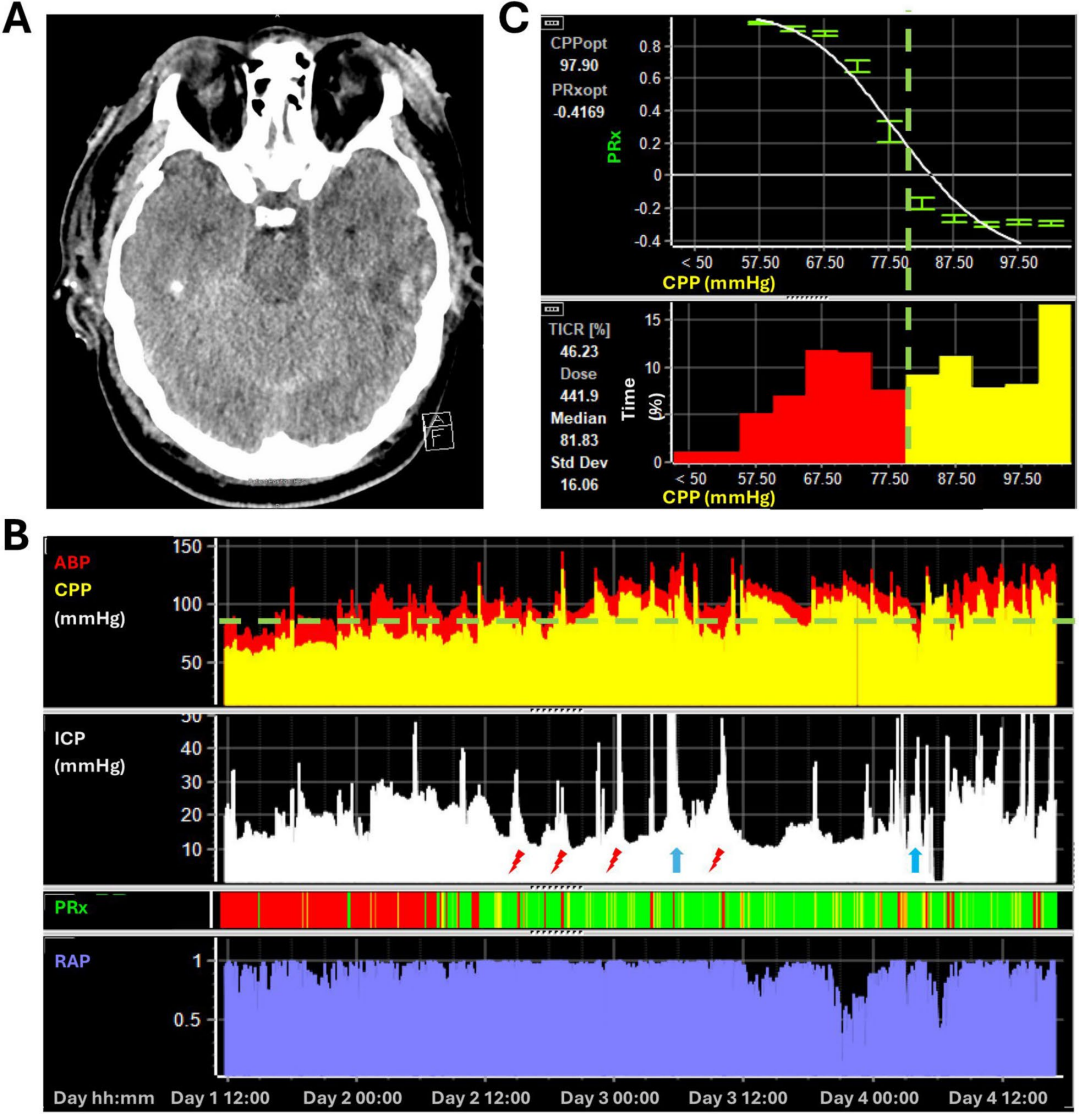
Monitoring trends and initial imaging of case 1. Panel **A** displays the initial CT scan with the intracranial hemorrhages. Panel **B** displays the MMM data. The minute-by-minute ABP, CPP, and ICP traces are shown in the upper panels. PRx is represented with a color-coded bar, where red indicates impaired and green indicates preserved vascular reactivity. The RAP, representing cerebrovascular compensatory reserve, is shown below. During the monitoring, the patient suffered multiple episodes with intracranial hypertension even in reaction to routine nursing procedures (marked using red bolts). Additionally, after an episode with high ICP (marked with the first blue arrow), the patient developed bilaterally non-reactive pupils. At the end of his stay, he developed refractory intracranial hypertension likely associated with infarction (as evidenced by the non-reactive pupils; marked with the second blue arrow). Panel **C** displays the relationship between CPP and PRx considering the full monitoring time and the frequencies of CPP values within the histogram below. The superimposed dashed lines in Panel **B** and **C** denote the CPP value representative of the LLR

A 25-year-old male patient was ejected through the windscreen of a van. Displaying an initial Glasgow Coma Score (GCS) of 3 (E1, V1, M1), the patient was intubated and brought to a District General Level Hospital. Hypertonic saline was administered to successfully reverse the initially bilaterally fixed, anisocoric pupils. The initial trauma imaging revealed severe extracranial injuries including a thoracic spine fracture, fractures of upper and lower extremities, lung contusions and a pneumothorax necessitating insertion of a chest drain. The initial cranial CT revealed contusions and a traumatic subarachnoid hemorrhage (Fig. 9A). Due to the severity of injury the patient was transferred to Addenbrooke’s Hospital. On arrival the patient was unstable with recurrent hypotensive episodes with systolic blood pressure dropping to 75 mmHg, ultimately stabilizing after transfusion of 7 units of packed red blood cells. 10 h after the initial injury (i.e. 3 h after admission), ICP monitoring commenced with a first reading of 26 mmHg. Although intracranial hypertension initially responded to treatment including deep sedation, ICP rose again above 20 mmHg within the first 6 h of monitoring, whereafter cooling using non-invasive temperature management (Arctic Sun) was initiated with a target of 34.5 °C. While hypothermia has not been shown to improve outcome when used early [61, 62] it has not been tested when later Tier therapy is required. That stated, hypothermia is often effective at reducing ICP, is part of our protocol, and its use is considered when Tier 2 therapies have failed (which is in itself congruent with the SIBICC Guidelines [63]). ICP rose to 30 mmHg around 12 h after initiation of ICP monitoring despite escalation of ICP management (including moderate hypothermia and metabolic suppression by means of deep sedation). Given the severity of the initial injury, DC was deemed unlikely to provide any significant benefit. Throughout the ICU course, the patient developed severe multi-organ failure and withdrawal of life sustaining therapies was initiated on day four.

**Fig. 10:**
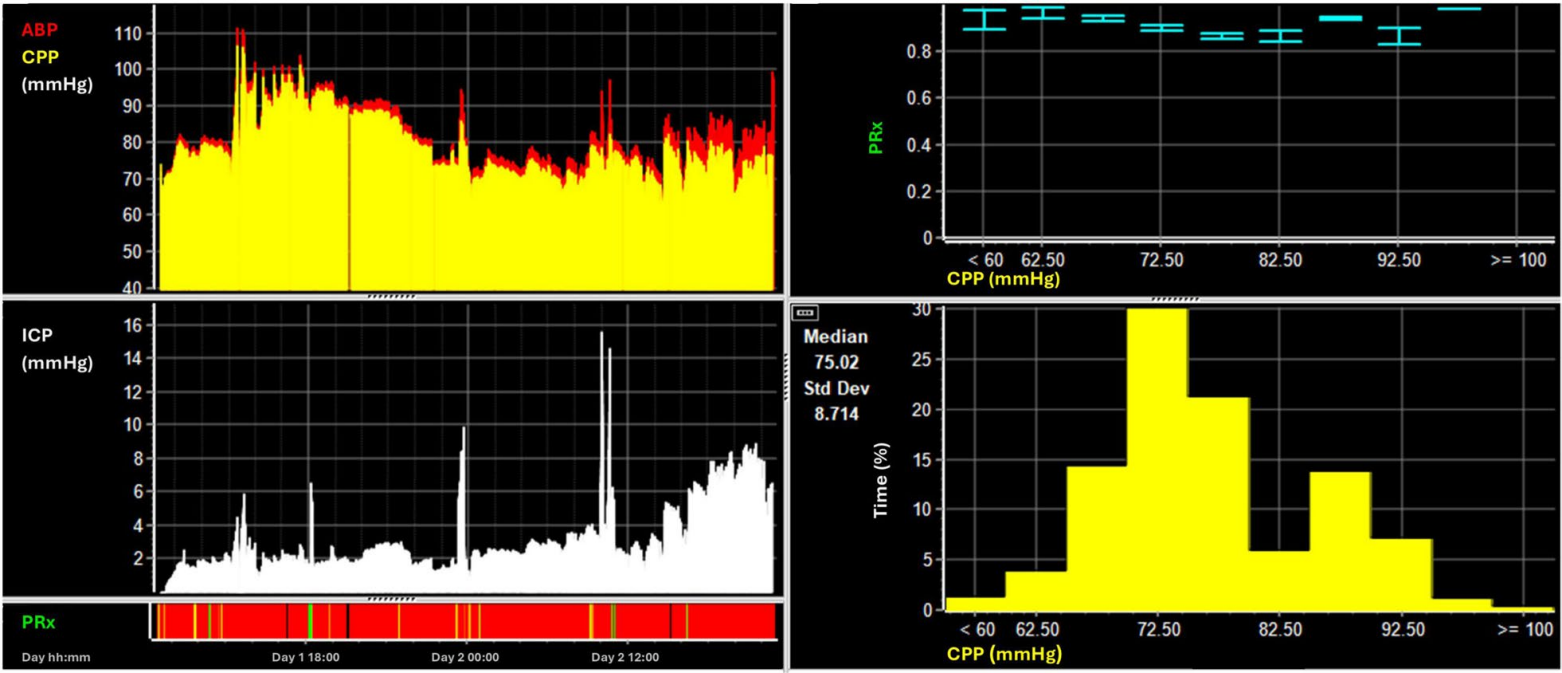
Red solid line—impaired cerebrovascular reactivity versus vasoparalysis. The “red solid line” pattern is characterized by a continuous episode of sustained increase in PRx lasting at least 30 min. In this figure, a second example of the red solid line pattern can be appreciated. The minute-by-minute ABP, CPP, and ICP trends are shown in the left upper panels. PRx is represented with a color-coded bar, where red indicates impaired vascular reactivity and green indicates preserved vascular reactivity. Initially identified as a predictor of sustained intracranial hypertension and brain death, the red solid line pattern has also been associated with episodes of cerebral hypoperfusion [13, 28]. In Fig. 9, this pattern occurred due to CPP remaining below the LLR for an extended period. In this case, no CPP value was associated with improved vascular reactivity despite a wide range of CPP values (from below 60 to over 100 mmHg) suggesting cerebral vasoparalysis. Consequently, neither CPPopt nor LLR could be identified. Clinically, this indicates that CPP-based optimization of vascular reactivity is unfeasible, and monitoring strategies should account for the passive (direct) relationship between changes in ABP and ICP

Owing to the highly dynamic and complex but brief stay of this patient, the MMM data offers valuable insights into various neurophysiological aspects (Fig. 9C). In this patient cerebrovascular reactivity was continuously assessed using PRx. Already upon admission to the ICU, the patient displayed impaired vascular reactivity. It is important to note that early PRx derangement does not necessarily reflect impaired brain perfusion. High PRx (i.e. PRx > 0.3) represents failure of cerebrovascular reactivity, the neuro-protective mechanism which should protect against changes in CPP. In this case, the PRx derangement can be interpreted as a consequence of the initial injury, but more importantly as a "risk factor" for secondary brain injury. A specific pattern displayed by this patient, a sustained increase in PRx—“red solid line” pattern [64], shows that the CPP was likely outside of the range of functioning reactivity for an extended duration. Without the added information derived from MMM, this case could not have been differentiated from a case of complete vasoparalysis (Fig. 10).

As we can appreciate from Fig. 9C, CPP was increased continuously from day 1 to day 3, from 60 to 70 mmHg (the generally accepted target range by international guidelines [17]) to 80 mmHg. In this patient, CPP was augmented according to CPPopt. In the figure, we can appreciate that PRx decreased with increasing CPP indicating improved autoregulation function, which was accompanied by a decrease in ICP, whereas routine methods did not result in significant improvements. The relationship between CPP and PRx was assessed considering the whole monitoring duration (Fig. 9B). A clear increase in PRx (i.e. worsening of cerebrovascular reactivity) can be appreciated below ~ 80 mmHg CPP (representing the LLR). During the first day, CPP remained below the LLR with consequently sustained deranged reactivity. Due to the retrospective nature of this analysis, it is unclear why earlier augmentation of CPP could not be achieved. It could be attributed to the initial cardiovascular instability. It is important to recognize that PRx merely describes the state of cerebrovascular reactivity, a mechanism which aims at protecting the brain. Failure of the mechanism does not per se reflect existing brain injury and functioning reactivity does not preclude the occurrence of secondary brain injury or unfavorable outcomes as was the case for this patient.

### Benefits of MMM

This case illustrates how MMM allows for the integration of different modalities (ICP, ABP), yielding otherwise hidden, clinically relevant metrics (e.g. PRx, LLR). Additionally, this case illustrates how MMM could support clinical decision making and personalization of treatment by providing insight into relevant pathophysiological mechanisms. Without the additional information derived, the origin of the red solid line pattern could not have been identified, and it would have been unclear whether CPP could be used to improve cerebrovascular reactivity and consequently ICP.

### Case 2: MMM in significant systemic hypoxia

**Fig. 11:**
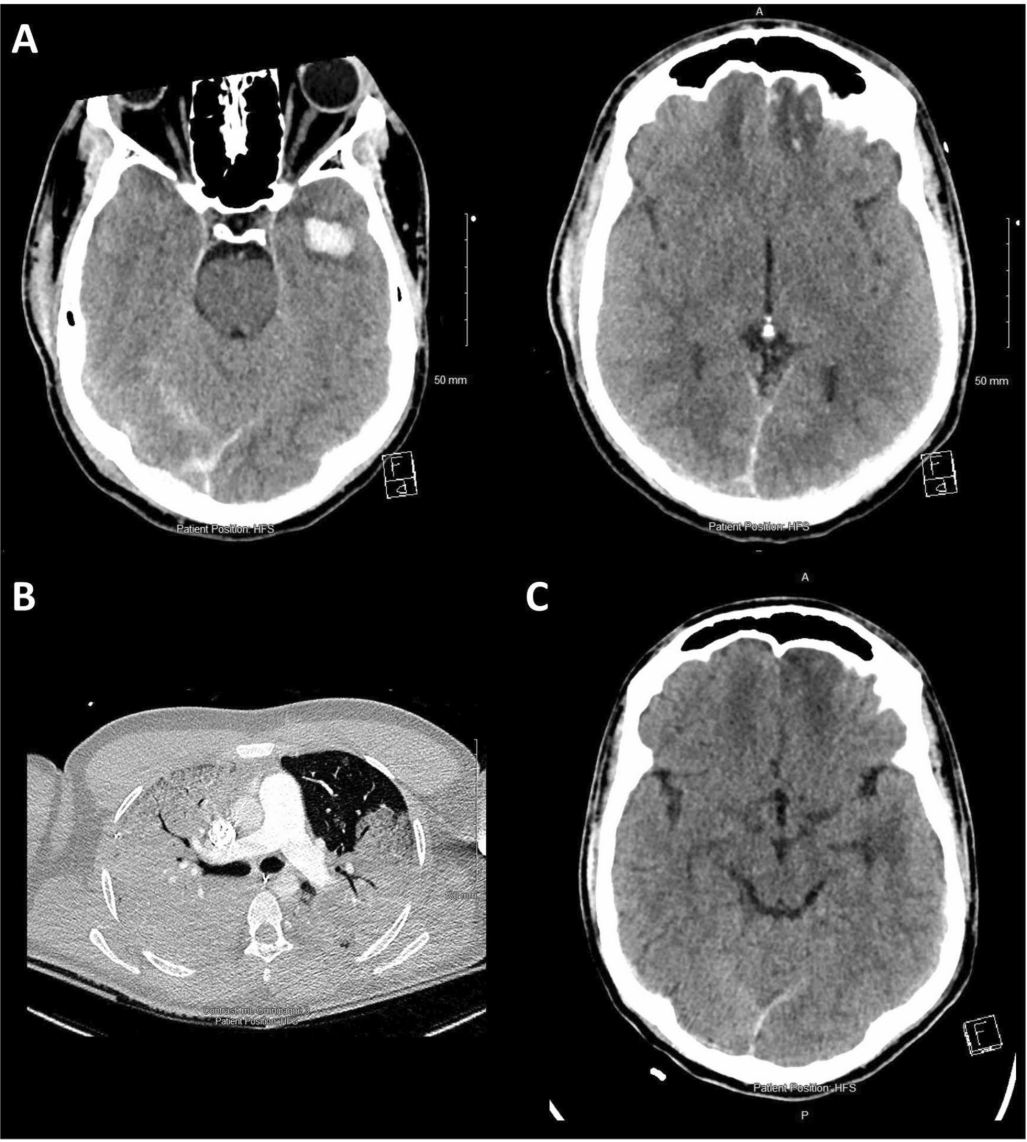
Imaging from case 2. Panel **A** shows the initial cranial CT scan with a frontotemporal contusion and a traumatic subarachnoid hemorrhage in addition to a parieto-temporal and base skull fractures. Panel **B** shows the thoracic CT scan displaying the extensive pulmonary consolidations and emboli. Panel **C** displays the imaging performed at the end of the hospital stay after cessation of extracorporeal membrane oxygenation. The hemorrhage seen on the initial imaging has been absorbed and only residual gliosis can be seen

A 25-year-old male presented with TBI following accidental fall out of the rear passenger window of a moving vehicle at 20 mph. The patient was intubated at the scene with a GCS of 8 (E1V1M6). An initial CT scan of the head revealed a frontotemporal contusion and a traumatic subarachnoid hemorrhage in addition to a parieto-temporal and base skull fractures (Fig. 11A). No extracranial injuries were identified. Given the severity of the injuries, ICP monitoring was initiated.

In the first three days following the injury, the patient's condition remained relatively stable, with the primary concern being ICP management. With deep sedation and the placement of an external ventricular drain, ICP was successfully kept within the target ranges. On day 4, the patient developed ventilator-associated pneumonia. Despite treatment with antibiotics, the patient required increasing levels of inspired oxygen fractions to maintain adequate oxygenation, prompting the clinical team to initiate PbtO2 monitoring. On day 6, a thoracic CT scan (Fig. 11B) revealed extensive pulmonary consolidations and emboli, which, in conjunction with the clinical appearance, fulfilled the criteria for acute respiratory distress syndrome.

**Fig. 12:**
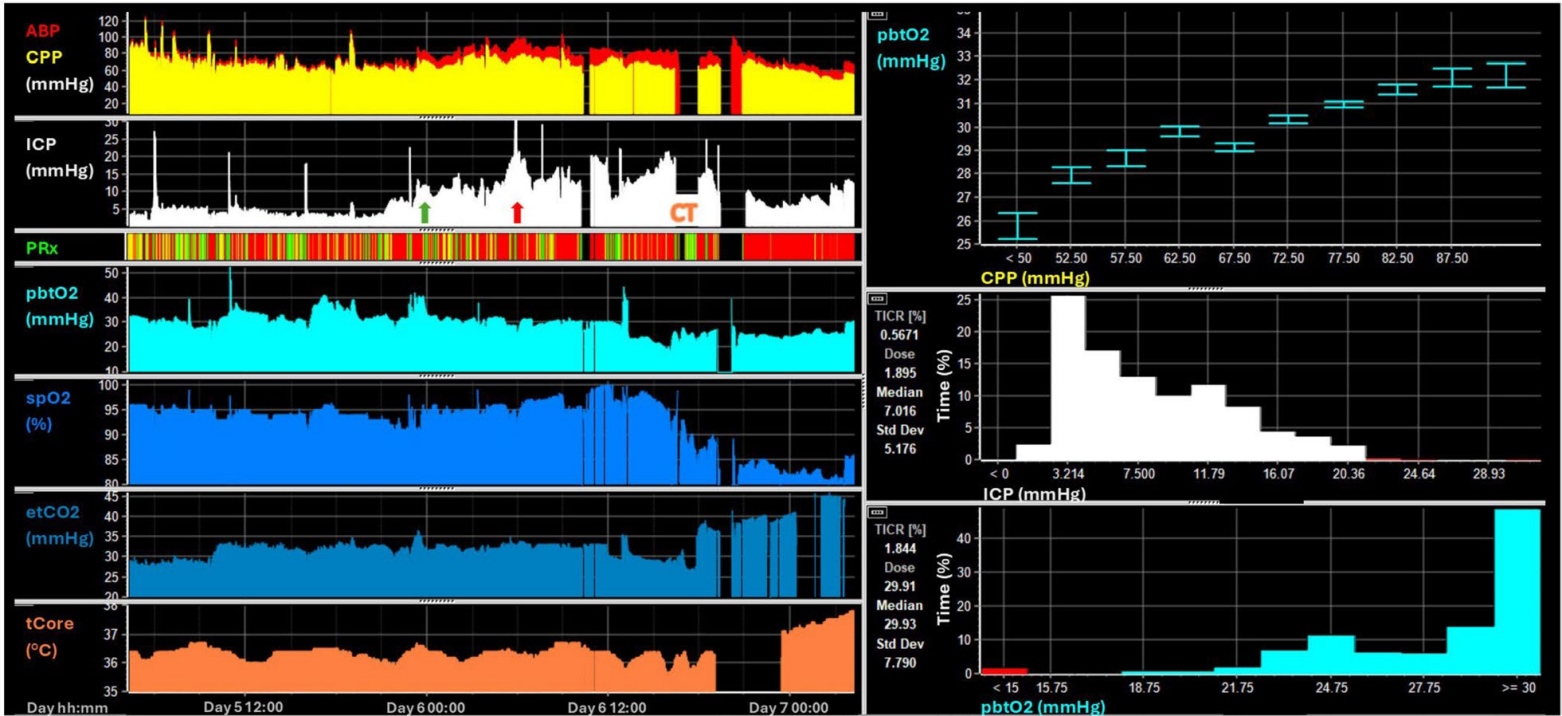
Multimodality data from case 2. The MMM data is displayed with minute-by-minute data on the left panels and derived doses and associations on the right. The green arrow displays the moment when sedation was reduced with an initial stable period of around 4 h before there was a secondary increase in ICP (marked with a red arrow). The distinct desaturation can be seen in the spO2 trace after the initiation of the thoracic CT. On the right top panel, the almost linear association between increasing CPP and PbtO2 can be seen. Below, the percentage time spent at the different ICP or PbtO2 levels are shown with only minimal time spent above an ICP of 20 mmHg and PbtO2 below 15 mmHg

During the CT scan, after being positioned flat, the patient desaturated. This episode can be appreciated within the MMM data (Fig. 12). A significant decrease in spO2 was observed around 13:00 on day 6, with levels dropping from approximately 95% to 80%. Moreover, there was an increase in core temperature starting from 36° and rising to above 38° in line with the acute respiratory distress syndrome caused by pneumonia. Standard management strategies did not result in improvement, leading to a discussion regarding initiation of extracorporeal membrane oxygenation given increasing evidence for its use after trauma [65]. The neurological status of the patient could not be assessed due to deep sedation for the purpose of ICP management and mechanical ventilation. However, despite the extreme respiratory compromise both ICP and CPP were consistently maintained within the acceptable limits. Additionally, PbtO2 remained stable at 30–35 mmHg, without dropping below 20 mmHg, even after systemic desaturation.

In terms of oxygen delivery to the brain, several factors must be considered. In this case, autoregulation was impaired throughout hospital stay, as highlighted by PRx. As a result, changes in CPP are directly translated into changes in cerebral blood volume and flow. The relationship between different CPP and PbtO2 levels (Fig. 12 top right) was almost linear, thereby confirming a pattern of conductive dependent oxygen delivery [66]. In addition, during the last 6 h of MMM shown in the figure, the decrease in CPP was counteracted by an increase in EtCO2. Assuming EtCO2 reflects paCO2 despite the presence of acute respiratory distress syndrome, this likely helped maintain CBF and PbtO2 because of the vasodilatory effect of CO2. The presence of preserved CO2 reactivity is confirmed by a concurrent increase in ICP with rising EtCO2. Of note, extended episodes of desaturation not only portray a danger for the brain, but also for the extracranial systems and encompass the risk of coagulopathy, acid–base imbalance and ischemia of the various organs. Considering the positive prognostic factors such as initial neurology, young age and imaging, no indications for relevant secondary brain injury within the MMM, and in view of profound systemic hypoxia, the decision to initiate extracorporeal membrane oxygenation therapy was made. Figure 11 panel C displays the imaging performed shortly before hospital discharge. The patient achieved full recovery and returned to their prior occupation 6 months after the TBI.

### Benefits of MMM

In this case, MMM was indispensable for assessing cerebral physiology and health in a patient inaccessible to clinical assessment, thereby supporting clinical decision making. This facilitated planning for advanced management with extracorporeal membrane oxygenation.

### Additional cases covered in the supplementary materials

Supplement B “The Autoregulation Curve and Secondary Brain Injury in Septic Shock,” presents MMM data from a patient who experienced septic shock following a TBI. This case underscores how MMM can reveal both early predictive indicators and the consequences of septic shock by examining data collected via TCD, PbtO2 monitoring, and microdialysis.

Supplement C “rSO2 Monitoring” illustrates a case involving secondary pneumocephalus. It demonstrates the advantages of integrating various monitoring modalities, including rSO2, to thoroughly elucidate the origin of dynamic ICP increases.

Supplement D “Cerebral Compensatory Reserve and Compliance in Decompressive Craniectomy” illustrates two cases, in which the assessment of compliance and compensatory reserve after DC allows for gauging the secondary ICP trajectory after DC.

## Discussion

In this case series we highlighted the advantages that the integration between high resolution based MMM combined with other modalities may provide to the treating clinician for understanding physiology and management of TBI patients admitted to intensive care. We provided detailed descriptions of MMM related key concepts and described their respective value within different pathophysiological scenarios.

In recent decades, neuromonitoring of TBI has evolved from relying solely on clinical evaluation to incorporating an increasing number of modalities for monitoring brain physiology. ICP was introduced in the 1960s by Lundberg before the establishment of specialized neurocritical care units[67]. Over the next 30–40 years, various additional treatment targets emerged, including the evaluation of CPP, brain oxygenation (PbtO2 and rSO2), and brain metabolism. The growing number of modalities and measures derived has increased the complexity of their integration and interpretation [9]. The application of these metrics has not only led to an increase in derangements of cerebral physiology diagnosed but also opened the door to numerous specific and non-specific therapeutic interventions. Despite the complexity, many measures are still presented as low-granularity data, often on separate devices. The growing field of neurocritical care bioinformatics [68] represents the key driver for the integration, processing, and temporal visualization of the different modalities allowing for further exploitation of the resulting data lakes. Integrating these modalities into a single monitoring tool may be crucial for improving patient care, advancing our understanding of brain physiology, and implementing more complex, data-driven methods [2, 7, 69, 70], as highlighted by this case series.

There are several limitations that must be acknowledged. As a retrospective analysis, this work is inherently limited in its ability to draw causal inferences. While MMM was used in the management of all cases, the exact rationale for specific clinical decisions cannot be fully elucidated retrospectively and the results do not directly prove bedside utility of MMM. The small number of cases, selected to highlight distinct scenarios, does not reflect the full spectrum of TBI patients, and the findings should be interpreted as illustrative rather than comprehensive. To date, the vast majority of evidence relies on retrospective descriptions, and some prospective randomized studies which failed to demonstrate the value for improving outcomes in patients with TBI. Key trials include the BEST TRIP trial [71] (which randomly assigned patients to have management based on or without ICP monitoring), the trial by Robertson et al. [72] (who randomly assigned patients to be managed according to ICP or CPP guided protocols), or most recently the OXY-TC trial [73] (which randomly assigned patients to receive care guided by ICP alone or ICP and PbtO2 monitoring). Within OXY-TC a post-hoc analysis suggested that the subset of patients with early high ICP had better outcomes with care guided by both ICP and PbtO2 monitors, and results from two currently running trials investigating care guided by ICP vs. ICP and PbtO2 monitoring (BOOST-3: NCT03754114 [74], and BONANZA: ACTRN12619001328167) are awaited.

This case-series emphasizes the physiological rationale and logical premise supporting its use, which is increasingly adopted in neurocritical care. Importantly, the utility of MMM relies on the clinician's ability to interpret multimodal data in a cohesive and integrated manner. The successful interpretation of MMM data in this series required interdisciplinary collaboration. While this collaborative approach is a strength, its feasibility in less specialized settings remains uncertain. This complexity underscores the need for ongoing training and experience to fully harness the potential of MMM. At times, even discordant or conflicting information from different monitoring modalities may arise, requiring significant expertise to identify which data may be flawed and why, an issue which potentially leads to errors or hasty decisions in a high-stress clinical environment such as an ICU. The role of MMM in these cases is best understood as part of a broader multimodal approach to decision-making rather than as an isolated determinant of success. Measures derived from NIRS, TCD, and microdialysis have been evaluated even less and are mostly limited to highly specialized centers and research. We abstained from describing additional neurophysiological measures such as EEG or electrocorticography. At our institution the full 10–20 EEG is used for routine (intermittent) monitoring and continuous EEG is only performed in specific instances (e.g. status epilepticus) even though such monitoring could reveal interesting patterns [75]. Processed EEG is only used for monitoring sedation, while electrocorticography is not routinely employed.

From a physiological standpoint, it is undeniable that MMM in TBI represents a significant advance over single modality neuromonitoring. Monitoring single measures such as ICP or PbtO2 provides limited insight and often lacks the necessary context for identifying the actual cause of injury or appropriate treatment options. MMM provides a more comprehensive overview and understanding of the dynamic and often complex changes which arise after such trauma. The integration of various modalities allows for the direct comparison of time trends and time-locked events. In this series, we presented the interplay and time profile of pathophysiological phenomena. This wide, albeit inexhaustive, range of cases, highlighted how MMM allows for deeper insights into cerebral physiology over and above any single monitoring metric. Our case reports should be seen as an excerpt of the various additional possibilities that arise with the use of MMM, rather than viewing the cases and events presented as the only reasons for using MMM. We hope this will encourage readers to undertake in-depth exploration of events found within their own patients.

## Supplementary Information

Supplementary materials 1. Supplement A: TBI ICP/CPP Management Algorithm.

Supplementary materials 2. Supplement B: CASE: rSO2 Monitoring.

Supplementary materials 3. Supplement C: CASE: The Autoregulation Curve and Secondary Brain Injury in Septic Shock

Supplementary materials 4. Supplement D: CASE: Cerebral Compensatory Reserve and Compliance in Decompressive Craniectomy.

## Data Availability

There is no additional data associated with this work.

## Abbreviations

ABP: Arterial blood pressure
CBF: Cerebral blood flow
FV: Cerebral blood flow velocity
CO2: Carbon dioxide
CPP: Cerebral perfusion pressure
CPPopt: Optimal CPP
DC: Decompressive craniectomy
EEG: Electroencephalography
EtCO2: End-tidal CO2
GCS: Glasgow coma scale
GOSE: Glasgow outcome scale extended
HR: Heart rate
ICU: Intensive care unit
LLR: Lower limit of reactivity
MMM: Multimodality neuromonitoring
NIRS: Near infrared spectroscopy
PRx: Pressure reactivity index
PbtO2: Brain tissue oxygenation
PSI: Pulse shape index
RAP: Compensatory reserve index
rSO2: Regional oxygen saturation
spO2: Blood oxygen saturation
TBI: Traumatic brain injury
TCD: Transcranial Doppler sonography
