# Supplementary material for: Unlocking the potential of high-resolution multimodality neuromonitoring for traumatic brain injury management: lessons and insights from cases, events, and patterns
Source: Crit Care. 2025 Mar 31;29:139. doi: 10.1186/s13054-025-05360-4 (PMC11956216; doi:10.1186/s13054-025-05360-4)

TBI ICP/CPP Management Algorithm

Local intracranial pressure and cerebral perfusion pressure management protocol. The specific treatment protocol has been described previously (Menon D, Ercole A. Critical care management of traumatic brain injury. Handbook of clinical neurology. 2017; 140:239-74).

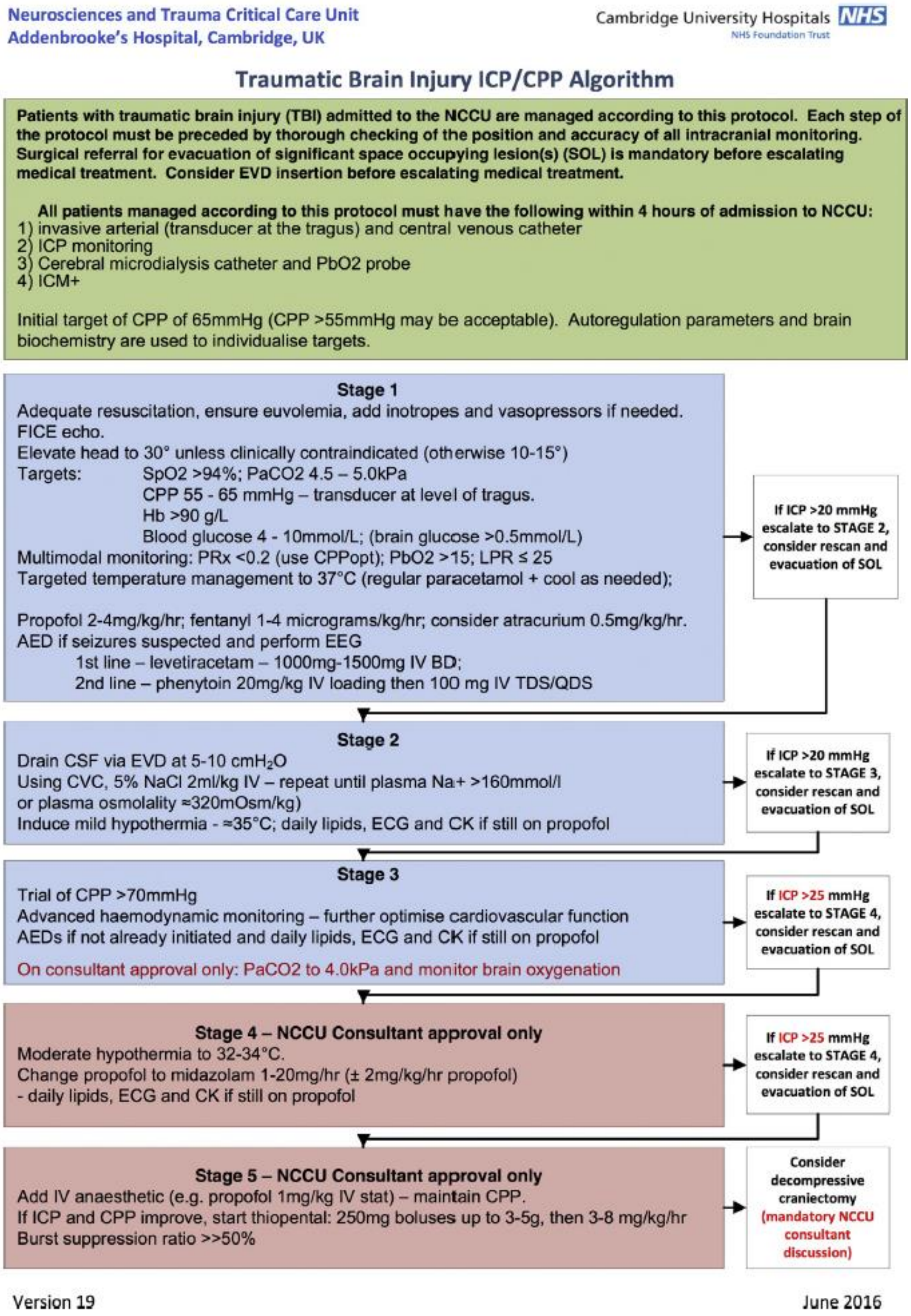

Supplement: Supplementary file 1 — Supplementary materials 1. Supplement A: TBI ICP/CPP Management Algorithm. [file 13054_2025_5360_MOESM1_ESM.pdf]
