# Supplementary material for: Unlocking the potential of high-resolution multimodality neuromonitoring for traumatic brain injury management: lessons and insights from cases, events, and patterns
Source: Crit Care. 2025 Mar 31;29:139. doi: 10.1186/s13054-025-05360-4 (PMC11956216; doi:10.1186/s13054-025-05360-4)
Supplement: Supplementary file 2 — Supplementary materials 2. Supplement B: CASE: rSO2 Monitoring. [file 13054_2025_5360_MOESM2_ESM.pdf]

CASE: rSO2 Monitoring

A 65-year-old female with a history of gait impairment and cognitive decline presented with severe TBI following a fall down a flight of stairs at her residence. The GCS at the scene was 8 (E1V2M5), and the patient initially had bilaterally sluggish yet reactive, isocoric pupils. During transport, the pupils became anisocoric (left pupil dilating to 4 mm and becoming unreactive). Imaging revealed a large left-sided subdural hematoma with a 5 mm left-to-right midline shift (Figure 1A). Hypertonic saline was administered, and the patient was transferred to Addenbrooke's Hospital for further monitoring and treatment. A craniotomy with hematoma evacuation was performed approximately 5 hours post-injury, followed by postoperative care on ICU. Postoperatively, pupils remained anisocoric, with the left pupil sluggishly reactive.

Figure 2 illustrates the MMM data for the first 24 hours after postoperative transfer to NCCU. Over approximately 12 hours, there was a gradual increase in ICP from about 12 to 20 mmHg. During the same period, hypertonic saline was given in repeated boluses to address intracranial hypertension (blue arrows). Despite the use of hypertonic saline and an escalation in sedation, ICP persisted at around 20–22 mmHg. In this context, NIRS monitoring provided additional insights. Immediately after ICU admission, rSO2 values were approximately 58% on the left side and 95% on the right. At around 18:00, during the rise in ICP, rSO2 values changed abruptly in conjunction with routine suctioning (i.e., a synchronous increase of ICP and ABP). Initially, there was a right-sided decrease in rSO2, followed by a subsequent contralateral increase. At approximately 04:00, both sides converged and stabilized at around 72%, with ICP remaining at about 20–22 mmHg. Although ICP had risen steadily overnight from around 12 mmHg, the clinical team deferred performing a CT scan until morning, given the relatively stable (though elevated) ICP. The follow-up CT obtained the next day (Figure 1B) showed a tension pneumocephalus, a known complication of subdural hematoma evacuation. In this case, pneumocephalus likely developed unilaterally before extending to the opposite side, eventually resulting in equalization of rSO2 values. The pneumocephalus shifted brain tissue away from the probe locations, causing readings reflective of extracranial soft tissue.

In this patient, ICP remained around 20 mmHg for the remainder of the hospital stay. A subsequent imaging study a few days after the diagnosis of pneumocephalus (Figure 1C) revealed a secondary thin subdural hematoma due to traction on bridging veins, as well as a hygroma. The patient eventually developed sepsis from ventilator-associated pneumonia and did not regain consciousness. Given the patient's frailty, recurrent prior falls, and neurocognitive decline, care was redirected after discussing the prognosis with surrogate decision makers on day 7 post-injury.

Benefits of MMM:

In this case, MMM clarified the etiology of the ICP elevation. At first glance, the paradoxical increase in left-sided rSO2 concurrent with the ICP increase might have led to a different interpretation, given that rising ICP typically reduces rSO2 rather than elevating it. The combined visualization of multiple monitoring modalities and their time trends made it possible to explore these events in detail.

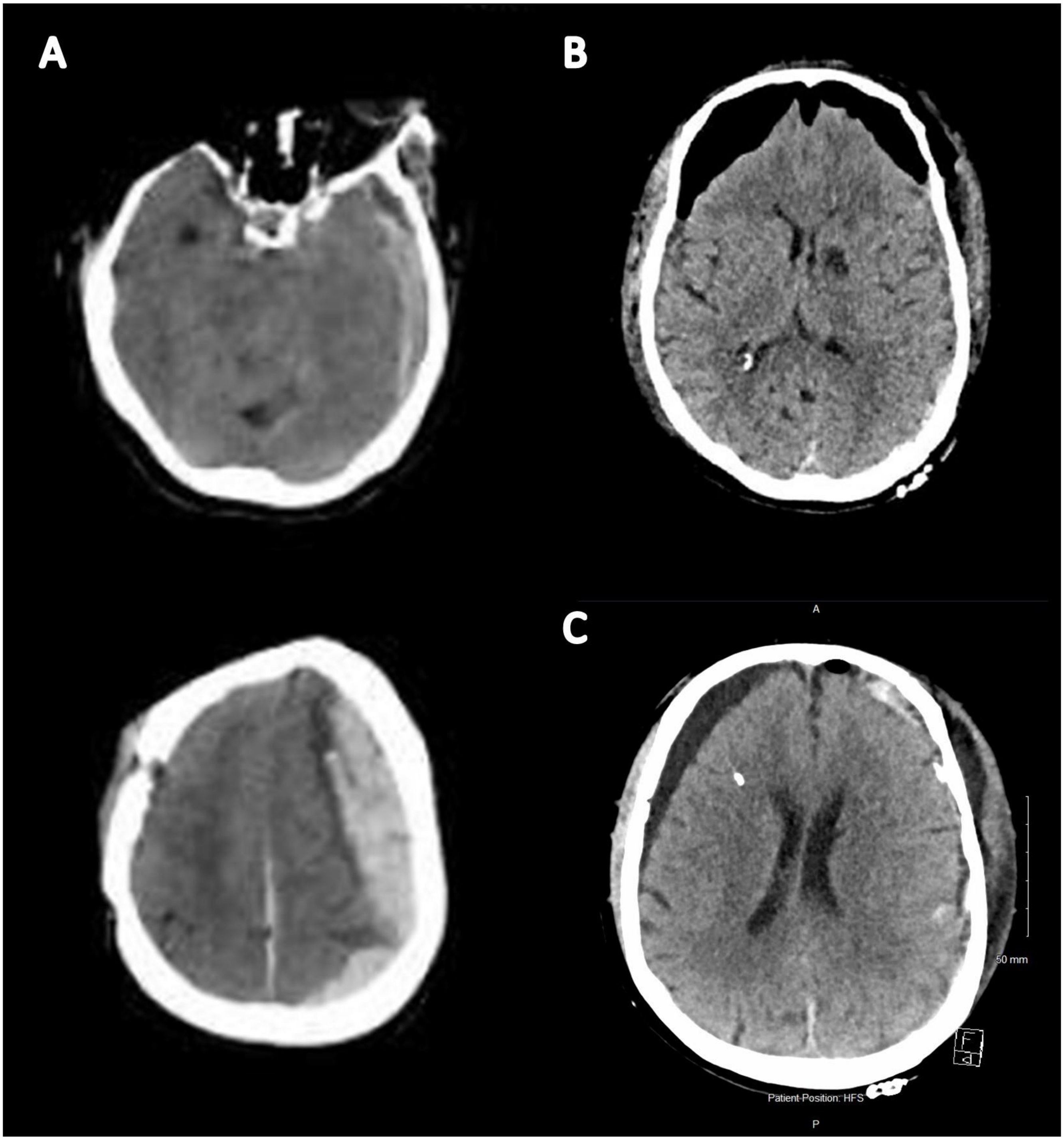

Figure 1. Cerebral imaging. Panel A shows the initial head CT displaying the subdural hematoma on the left. Panel B displays the CT performed after the increase in intracranial pressure – marked in figure 16 – which showed the extensive bilateral pneumocephalus. The last CT (performed a few days after the diagnosis), shown in panel C, shows a secondary thin subdural hematoma on the left and a hygroma on the right.

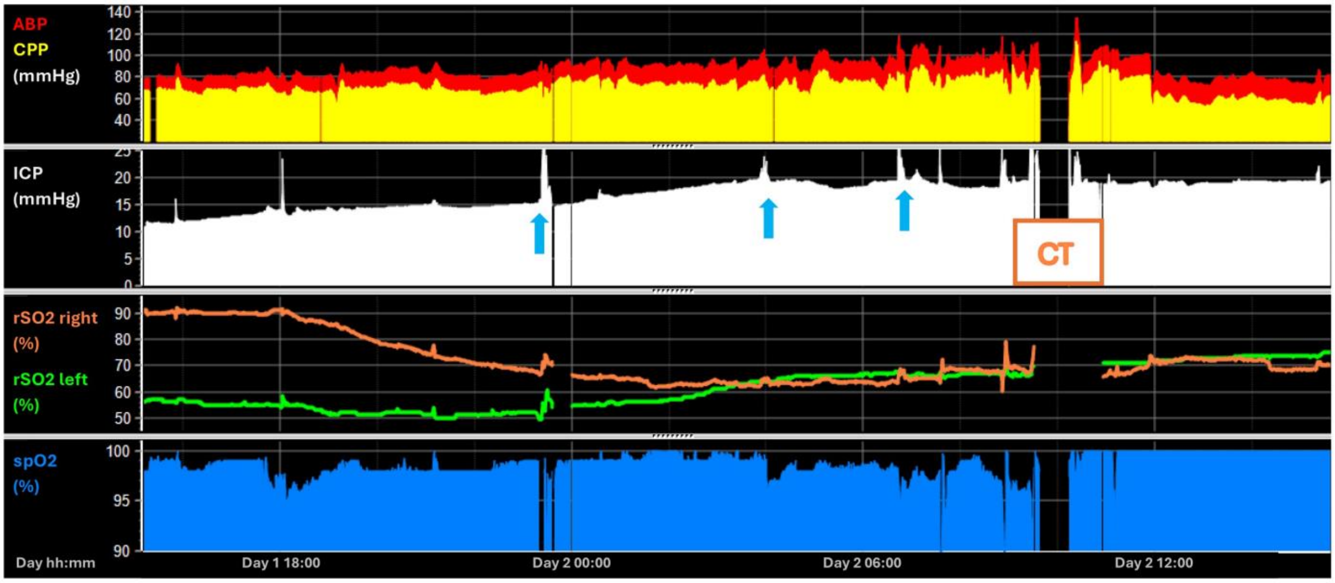

Figure 2. Multimodality monitoring data. The time trends of ABP, CPP, and ICP are displayed in on the top. The superimposed blue arrows indicate the time points at which hypertonic saline was administered. Below the right and left rSO2 time trends can be appreciated. ICP rose from around 12 mmHg to around 20-22 mmHg overnight. During the same time, both rSO2 values equalized around 5 hours before the CT was performed.
