# Supplementary material for: Unlocking the potential of high-resolution multimodality neuromonitoring for traumatic brain injury management: lessons and insights from cases, events, and patterns
Source: Crit Care. 2025 Mar 31;29:139. doi: 10.1186/s13054-025-05360-4 (PMC11956216; doi:10.1186/s13054-025-05360-4)
Supplement: Supplementary file 3 — Supplementary materials 3. Supplement C: CASE: The Autoregulation Curve and Secondary Brain Injury in Septic Shock [file 13054_2025_5360_MOESM3_ESM.pdf]

CASE: The Autoregulation Curve and Secondary Brain Injury in Septic Shock

A 25-year-old male patient involved in a rollover road traffic collision. He was intubated at the scene due to agitation and subsequently transported to our hospital. The initial GCS was 7 (E1, V2, M4). The initial cranial CT showed a right sided subdural hematoma, and multiple intraparenchymal hemorrhages and contusions (Figure 1A). The patient had also sustained a right sided pulmonary contusion and a liver laceration. Invasive neuromonitoring was established including assessments of ICP, PbtO2, and cerebral microdialysis.

Following an initial stable period of around 30 hours, the patient developed sinus tachycardia starting on day 2 after the initial injury, necessitating increasing norepinephrine dosages to maintain the target CPP of 65 mmHg (Figure 1B). At the onset of the recording, which started around 30 hours post admission, ABP, CPP and ICP were within the target ranges. However, the patient already displayed tachycardia, with an initial HR of 125-130 beats per minute. Starting from around 13:00 there was a distinct increase of HR accompanied by a distinct sustained sympathovagal imbalance evidenced by a decrease in low frequency high frequency ratio of HR variability. In patients who subsequently develop shock, this HR variability metric has been reported to decrease, correlating with increased cardiac vagal activity and reduced sympathetic drive(1). In addition, a gradual rise in EtCO2 is observed despite continuous moderate hyperventilation aimed at controlling the rise in $PaCO_2$ and, consequently, ICP. In this case, the increasing EtCO2 likely reflects an elevated metabolic rate. The combination of increasing HR, decrease in low frequency high frequency ratio of HR variability, and a rising metabolic rate were interpreted as indicators of impending cardiovascular instability, prompting further investigation. Bedside echocardiography supported a low intravascular filling state, demonstrating decreased cardiac output, reduced left ventricular size, and a persistently collapsed inferior vena cava despite fluid challenge. Blood gas analysis revealed a rise in lactate level from 0.72 mmol/l to 3.71 mmol/l consistent with both reduced intravascular volume and increased metabolic demand. As the patient was already receiving high doses of norepinephrine, vasopressin was added to augment peripheral vasoconstriction. In parallel with cardiovascular decompensation, the patient developed escalating temperatures, necessitating high fractions of inspired oxygen to maintain a targeted spO2 of 94% and a pO2 exceeding 10 kPa. Despite these interventions, from 17:00 onward, progressive hemodynamic compromise coincided with a decrease in cerebral perfusion pressure below 20 mmHg (Figure 1B, orange arrow). Subsequent imaging findings and elevated inflammatory markers (C-reactive protein 168 mg/L) alongside neutrophilia confirmed the diagnosis of septic shock, likely secondary to pneumonia. Although sputum cultures did not yield a specific pathogen, this may be attributable to the patient's existing antibiotic regimen of co-amoxiclav and vancomycin, which had been initiated for presumed community-acquired pneumonia and a skin and soft tissue infection.

In addition to standard monitoring, this patient underwent daily continuous TCD assessments, which included the episode described. These extended TCD measurements revealed a noteworthy physiological phenomenon: the cerebral autoregulatory curve (Figure 1C). Typically, only a portion of this curve can be demonstrated in individual patients; however, the shock-related wide range of CPP values allowed for observation of a broader spectrum. Below the lower breakpoint, CBF declines in proportion to CPP, substantially increasing the risk of secondary brain injury through hypoperfusion and hypoxia. Although hypotension lasted for only about 40 minutes, its impact on cerebral oxygenation persisted significantly longer, despite peripheral oxygen saturation remaining above 92–94% (Figure 1D). rSO2 displayed a marked

and sustained decrease for nearly six hours. Concurrently, microdialysis highlighted the metabolic ramifications of this event, with an increase in the lactate-to-pyruvate ratio from 20 to 47, indicative of a shift to anaerobic metabolism. Reduced blood flow led to a transition from oxidative phosphorylation to anaerobic glycolysis, reflected by rising lactate levels from 1.7 to 3.0 µmol/L once glucose had been largely depleted (dropping from 1.8 to 0.2 µmol/L). A corresponding increase in glycerol concentration (from 55 to 132 µmol/L) was also noted, suggesting ongoing cell degradation and, specifically, membrane breakdown.

On the following day, the patient's condition deteriorated. Although ICP remained controlled, sedation reduction and neurological assessment could not be performed due to the onset of pneumonia and acute respiratory distress syndrome. The patient subsequently developed fulminant multi-organ dysfunction and withdrawal of life-sustaining therapies was undertaken five days post-injury.

Benefits of MMM:

In this case, MMM highlighted both predictive indicators (including changes in HR variability and elevated metabolic rate) and the ramifications of septic shock, particularly its prolonged effects on cerebral oxygenation and metabolism. Given the complexity of cerebral metabolism following TBI, the use of multiple parameters such as FV and microdialysis-derived lactate-to-pyruvate ratio and glycerol enable clinicians to identify pathophysiological disturbances and their detrimental consequences. If any of these monitoring techniques had been employed in isolation, neither the early warning signs nor the subsequent derangements could have been reliably interpreted.

1. Chen WL, Kuo CD. Characteristics of heart rate variability can predict impending septic shock in emergency department patients with sepsis. Academic emergency medicine. 2007;14(5):392-7.

Figure 1. Multimodality monitoring data and imaging. Panel A displays the initial CT scan with the intracerebral hemorrhages displayed on the left image and the subdural hematoma on the right image. Panel B depicts the 12-hour section during which the septic shock occurred. On the top, ABP and CPP are displayed with a distinct decrease after 17:00 representing the septic shock (marked with an orange arrow in all panels). Below the changes in HR, HR variability and EtCO2 are shown. The distinct increase in HR, decrease in HR variability and secondary increase in EtCO2, marked using red arrows, were seen as indicators of impending cardiovascular instability. Panel C displays the relationship between CPP and FV – i.e. the autoregulation curve. The lower limit of autoregulation is marked using a dashed green line both on the autoregulation curve as well as the minute-by-minute CPP trend. Panel D displays the secondary brain injury with decreases in rSO2 (top) and metabolic derangements (bottom). The time-points of either microdialysis assessment is shown using a blue star in panel B.

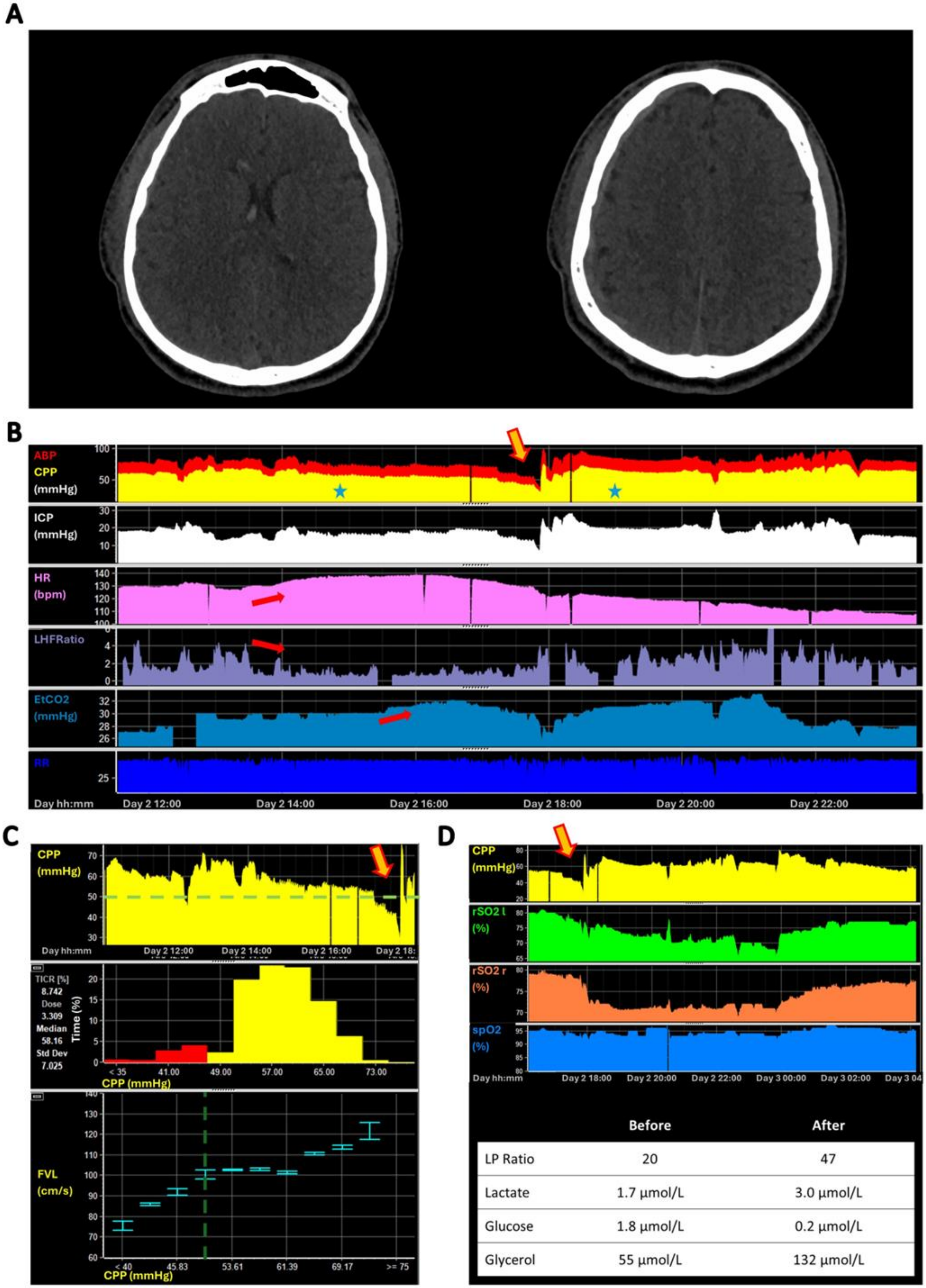

	Before	After
LP Ratio	20	47
Lactate	1.7 µmol/L	3.0 µmol/L
Glucose	1.8 µmol/L	0.2 µmol/L
Glycerol	55 µmol/L	132 µmol/L
