# Supplementary material for: Unlocking the potential of high-resolution multimodality neuromonitoring for traumatic brain injury management: lessons and insights from cases, events, and patterns
Source: Crit Care. 2025 Mar 31;29:139. doi: 10.1186/s13054-025-05360-4 (PMC11956216; doi:10.1186/s13054-025-05360-4)
Supplement: Supplementary file 4 — Supplementary materials 4. Supplement D: CASE: Cerebral Compensatory Reserve and Compliance in Decompressive Craniectomy. [file 13054_2025_5360_MOESM4_ESM.pdf]

CASE: Cerebral Compensatory Reserve and Compliance in decompressive craniectomy

The following description includes two patients who underwent secondary DC.

Case A. A 35-year-old male suffered a severe TBI secondary to a road traffic collision involving a motorcycle and a car. He had a GCS of 7 (E1V2M4) and was intubated at the scene. An initial CT scan of the head revealed an extensive left cerebral subarachnoid hemorrhage, a shallow left subdural hematoma, and possible contusions of the left inferior frontal and temporal lobes (Figure 1). Additionally, fractures to the facial bones, ribs, and extremities were noted. Given the severity of the injuries, a multi-channel cranial access device ("triple-bolt") was placed to monitor ICP, PbtO2, and cerebral metabolism.

After an initial stable period, ICP started to rise 16 hours after ICU admission. After a short-lasting improvement with increased sedation and muscle relaxation (midazolam, propofol, fentanyl, atracurium) for 16 hours, non-invasive temperature management (Arctic Sun) was initiated with an initial target of 35.5°C at 30 hours after admission, 34.5°C at 47 hours after admission and 34°C at 51 hours after admission. Despite escalating ICP management strategies, including therapeutic hypothermia (34°C), paralysis and deep sedation (to induction of burst-suppression), hyperosmolar therapy (hypertonic saline), and moderate hyperventilation (pCO2 3.8-4.3kPa), the ICP remained elevated at 20-24 mmHg (Figure 2A). A follow-up CT scan performed on the same day showed hemispheric swelling, expansion of the contusions with cisternal compression and a slight midline shift of less than 5 mm. Optimization of CPP based on PRx did not significantly reduce ICP. To maintain ICP near the target range, hypertonic saline and mannitol were administered frequently, albeit with limited success. As a result, a secondary left sided decompressive hemicraniectomy was performed around 60 hours after ICU admission. Post-intervention, there was a clear improvement. The CT performed after the DC revealed a well-positioned extensive skull defect with evidence of outward bulging of the brain parenchyma through the craniectomy window, consistent with ongoing cerebral edema (Figure 1A). No new areas of intracranial hemorrhage or infarction could be identified. Temperature was normalized, and both paralysis and sedation could be weaned within days after DC. Outcome was favorable, with a GOSE of 6 at 6 months.

Case B. The second case involved a 45-year-old male who experienced a sports-related accident without loss of consciousness. Following the incident, the patient suffered from repeated episodes of vomiting, headache, and dizziness. Failing to improve, the patient was prompted by friends to go to the emergency room as a walk-in patient. Upon arrival, the patient had become confused with subsequent neurological deterioration to GCS 10 (E3V2M5) while in the emergency room. An initial CT scan (Figure 1B) revealed a large left temporal and bifrontal contusions, and a thin left subdural hematoma. Additionally, there was a skull fracture along the right parietal bone extending to the skull base, with a small amount of intracranial air. After initially being admitted to a high dependency unit, the patient became increasingly agitated and disoriented. The neurological deterioration was associated with arterial hypertension (ABP of 200/90 mmHg) suggestive of Cushing reflex in the context of intracranial mass effect. The airway was secured with an endotracheal tube and organ support was provided in the ICU. A triple-bolt was placed for monitoring. ICP started to rise 17 hours after ICU admission, whereafter the decision for increasing sedation to include midazolam, propofol, fentanyl and for administering muscle relaxants continuously (atracurium) was made. 27 hours after ICU admission, despite deep sedation, ICP continued to rise whereafter cooling using non-invasive temperature management (Arctic Sun) was initiated with an initial target of 35°, which was then decreased to 34° 44 hours later. Despite

intensified treatment, including deep sedation, multiple administrations of hypertonic saline (Na 159 mmol/L), paralysis, and active cooling (below 35°C), ICP continued to rise. Due to refractory intracranial hypertension a secondary left sided decompressive hemicraniectomy was performed 63 hours after ICU admission (Figure 2B). The initial ICP reading post-DC was 14 mmHg. The CT-imaging acquired after the DC can be found in figure 1B with demarcation of the left frontal and temporal infarctions as well as a residual compression of the left lateral ventricle. However, unlike the prior case, ICP began to increase again shortly after the DC leading to an extended period of high intensity therapy and a long ICU stay. At 6 months the patient reached a GOSE of 4.

Figure 2 shows an excerpt of the MMM of either patient including the sections directly before and after secondary DC. In both cases, the immediate effect of the DC on ICP can be seen with resulting values within the single digit range. However, while in case A ICP was stabilized for an extended period, in case B ICP started rising continuously leading to a worsening of cerebral oxygenation (PbtO2). One striking difference concerns the compensatory reserve (as evaluated using RAP – the correlation coefficient between ICP amplitude and mean ICP). After DC, the compensatory reserve was increased effectively in patient A (values closer to 0 represent better compensatory reserve), while the effect in patient B was minimal with values between 0.8 and 0.9. Additionally, compliance, estimated using the PSI improved from values around 3 to 2 (with higher values being more pathological) in patient A, while there was a distinct increase from PSI 3 to 4 despite the DC in patient B. Consequently, based on the information derived from the MMM, the clinicians decided to wean ICP targeted therapies more aggressively in case A, while they remained cautious in case B, even though ICP was reduced to physiological ranges in either case.

Benefits of MMM:

While ICP monitoring forms the basis of clincial decision algorithms in neurointensive care, in this case, MMM and the acquisition of high-resolution waveform level data necessary for the PSI estimation allowed for gauging the trajectory of the patients after DC by revealing the compensatory reserve in absence of differences in ICP.

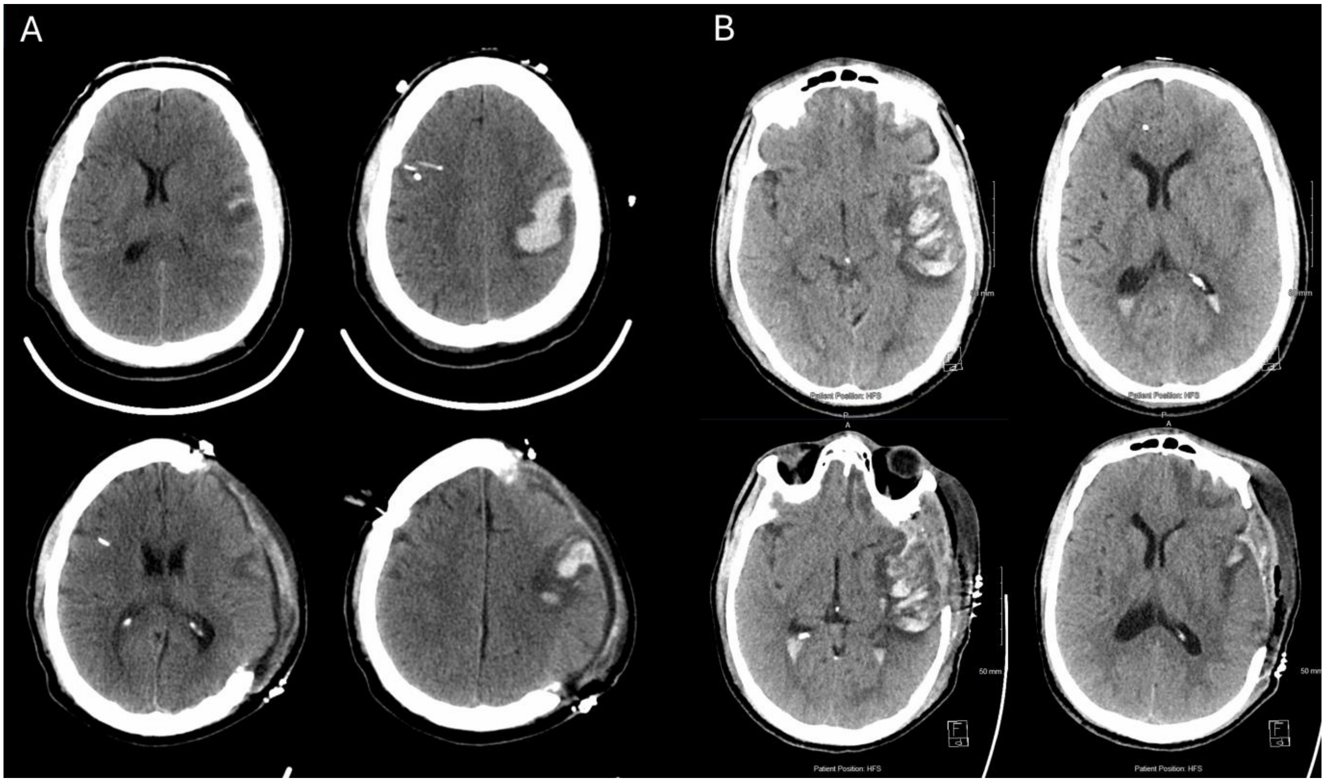

Figure 1. Cerebral imaging before and after decompressive craniectomy. The CT images prior (top panels) and post (bottom panels) decompressive craniectomy for case A (panel A) and B (panel B) are displayed. In the top images of case A, the expanded contusion with perihematomal edema can be appreciated. In the top images of case B, the extensive frontal and temporal contusions can be appreciated.

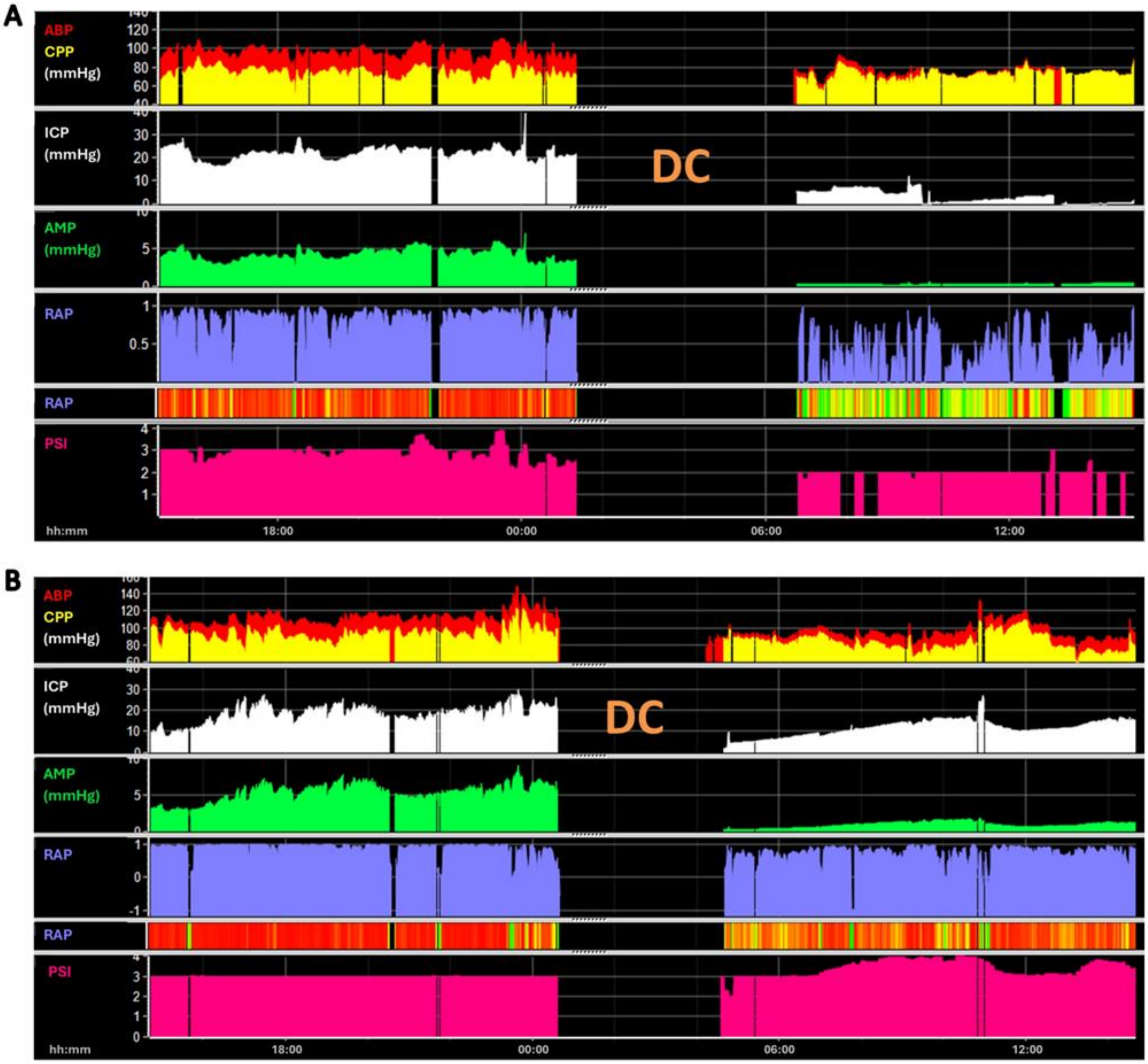

Figure 2. Multimodality data. The figure shows multimodality monitoring screenshots for the two patients in case A (panel A) and B (panel B), displaying minute by minute time trends of ABP, ICP, CPP, ICP amplitude (AMP), the compensatory reserve index RAP, and the pulse shape index PSI. RAP is also displayed with a color-coded risk bar with the threshold for the color change from green to red at a value of 0.6. While there was an improvement in either metric in patient A after DC, there was no such improvement in patient B despite the decrease in absolute ICP value.
